# Transcriptomics reveals a core transcriptional network of K-type cytoplasmic male sterility microspore abortion in wheat (*Triticum aestivum *L*.*)

**DOI:** 10.1186/s12870-023-04611-2

**Published:** 2023-12-06

**Authors:** Baolin Wu, Yu Xia, Gaisheng Zhang, Yongqing Wang, Junwei Wang, Shoucai Ma, Yulong Song, Zhiquan Yang, Lingjian Ma, Na Niu

**Affiliations:** 1 College of Agronomy, Northwest A & F University, Key Laboratory of Crop Heterosis of Shaanxi Province, Wheat Breeding Engineering Research Center, Ministry of Education, Yangling, 712100 Shaanxi China; 2 School of Agriculture and Biology, Shanghai Jiao Tong University, Shanghai, 200240 China

**Keywords:** Cytoplasmic male sterility, Transcriptome sequencing, Plant hormone, MAPK pathway, Spliceosome, Wheat

## Abstract

**Background:**

Cytoplasmic male sterility (CMS) plays a crucial role in hybrid production. K-type CMS, a cytoplasmic male sterile line of wheat with the cytoplasms of *Aegilops kotschyi*, is widely used due to its excellent characteristics of agronomic performance, easy maintenance and easy restoration. However, the mechanism of its pollen abortion is not yet clear.

**Results:**

In this study, wheat K-type CMS MS(KOTS)-90-110 (MS line) and it’s fertile near-isogenic line MR (KOTS)-90-110 (MR line) were investigated. Cytological analysis indicated that the anthers of MS line microspore nucleus failed to divide normally into two sperm nucleus and lacked starch in mature pollen grains, and the key abortive period was the uninucleate stage to dinuclear stage. Then, we compared the transcriptome of MS line and MR line anthers at these two stages. 11,360 and 5182 differentially expressed genes (DEGs) were identified between the MS and MR lines in the early uninucleate and binucleate stages, respectively. Based on GO enrichment and KEGG pathways analysis, it was evident that significant transcriptomic differences were “plant hormone signal transduction”, “MAPK signaling pathway” and “spliceosome”. We identified 17 and 10 DEGs associated with the IAA and ABA signal transduction pathways, respectively. DEGs related to IAA signal transduction pathway were downregulated in the early uninucleate stage of MS line. The expression level of DEGs related to ABA pathway was significantly upregulated in MS line at the binucleate stage compared to MR line. The determination of plant hormone content and qRT-PCR further confirmed that hormone imbalance in MS lines. Meanwhile, 1 and 2 DEGs involved in ABA and Ethylene metabolism were also identified in the MAPK cascade pathway, respectively; the significant up regulation of spliceosome related genes in MS line may be another important factor leading to pollen abortion.

**Conclusions:**

We proposed a transcriptome-mediated pollen abortion network for K-type CMS in wheat. The main idea is hormone imbalance may be the primary factor, MAPK cascade pathway and alternative splicing (AS) may also play important regulatory roles in this process. These findings provided intriguing insights for the molecular mechanism of microspore abortion in K-type CMS, and also give useful clues to identify the crucial genes of CMS in wheat.

**Supplementary Information:**

The online version contains supplementary material available at 10.1186/s12870-023-04611-2.

## Introduction

 Increasing crop yields in a sustainable and efficient manner is critical to meeting the growing global demand for food [1]. Wheat (*Triticum aestivum* L.), as one of the most important crops in the world, delivers around 20% of our food calories and protein, and feeds nearly 40% of the world’s population [2, 3]. Wheat has obvious heterosis in many aspects such as yield and adaptability, and efficient heterosis utilization in wheat also offers one of the most effective strategies for improving the yield of this crop [4]. There are four major systems have been utilized in studying heterosis and producing commercial hybrid wheat, which is classified into two categories: heritable male sterility and physiological male sterility [5]. Of heritable male sterility, Cytoplasmic male sterility (CMS) is caused by mitochondrial genes together with nuclear genes, which leads to abnormal anther development and pollen abortion [6, 7]. Up to now, more than 130 nuclear and cytoplasmic hybrids were observed, including T, K, V, D, A, or P type CMS [8]. CMS is a very powerful tool for hybrid wheat breeding, but the mechanism responsible for pollen abortion in CMS remains unclear at present. K-type CMS lines with *Aegilops kotschyi* cytoplasm showed stable fertility and was easy to restore, so it is widely studied and used [9]. Most CMS-determine genes were derived from the rearrangement of mitochondrial genome segments characterized as a chimeric open reading frame (ORF) [10]. The products of these CMS-determine ORFs are cytotoxic and resulted in dysfunction of mitochondria by impairing Adenosine-Triphosphate (ATP) synthesis, programmed cell death (PCD), cell-death signaling or Reactive Oxygen Species (ROS) production [11]. Genetically speaking, common wheat is an allohexaploid species, and this plant has a large complex genome, which is 50 times larger than rice [3]. Male reproductive processes occur within the anther. and diploid sporogenous cells go through meiosis to form haploid microspores, which eventually develop into pollen grains or the male gametophyte [12]. Anther development is a complex process involving numerous genomes and multiple metabolic pathways in cells, and any disorder during anther development can lead to pollen abortion, and then result in male sterility [13, 14].

Comparative transcriptome analysis provides a powerful high-throughput method for identifying key genes involved with male sterility pathways and for illuminating the pertinent molecular mechanisms [15, 16]. Plant hormones have strong effects on anther’s fertility [17]. Disruption of genes associated with hormone biosynthesis and signal transduction pathway leads to abnormal anthers or sterile pollen [18]. For example, in barley (*Hordeum vulgare* L.), the decrease of IAA content in anthers will lead to pollen abortion [19]; ABA and IAA are involved in the PCD (programmed cell death) of petunia (*Petunia hybrida* L.) microsporocytes at the meiosis stage [20]; transcriptome and hormone comparison analysis discovered that the microspore abortion of cytoplasmic male sterile *Brassica napus* lines could relate to the excessive ABA content in the anthers [21] (The mitogen-activated protein kinase (MAPK) cascade is not only involved in biotic and abiotic stress responses but also participates in the regulation of plant development [22, 23]. Transcriptomic analysis discovered that MAPK signal transduction pathway participate in fertility conversion in thermosensitive genetic male sterility line Zhu1S rice under high temperature [17]. *OsMAPK6*, as an important member of the MAPK cascade, affects male fertility by reducing microspore number and delaying tapetum degradation in rice (*Oryza Sativa* L.) [24]. Alternative splicing (AS) is an important regulatory process in eukaryotes, which can use different splicing sites to produce multiple mRNA isoforms from the same pre-mRNA, thus increasing transcriptomic and proteomic diversity to regulates plant development and stress responses [25, 26]. There are five basic types of AS events, namely, exon skipping (ES), intron retention (IR), alternative 5’ splice sites (A5SS), alternative 3’ splice sites (A3SS), and mutually exclusive exons (MEs). The proportion of AS events varies in different species: IR is the most common AS event in plants, while ES is the most prevalent one in animals [27]. AS participates in the regulation of multiple pathways during plant growth and development. Previous research has found that IR is a stage-specifc mechanism of functional attenuation of a subset of co-regulated, functionally related genes during early stages of pollen development [28]. Therefore, the analysis of AS in wheat anthers is of great significance to describe the mechanism of pollen development. Transcriptomic analysis discovered that alternative splicing plays an important regulatory role in tomato pollen in response to heat stress [29]. In addition, sucrose and starch are accumulated as energy reserves and carbon skeleton sources, and they are necessary for pollen and anther development in the later mature developmental stages [30]. Futhermore, “flavonoid biosynthesis metabolism” [31], “phenylpropanoid biosynthesis” [30] and “lipid metabolism” [32] all play significant roles in the development of plant pollen.

We have previously used the K-type CMS wheat MS(KOTS)-90-110 (MS line) as the female parent in crosses with other high-quality wheat varieties. Strong and dominant hybrid wheat combinations are produced by this line due to its benefits of stable sterility, a variety of recovery sources, and a low phenotypic coefficient of variation in the F1 generation [33]. The K-type cytoplasmic male sterile line (MS line) was crossed with restorer lines with high restoring degrees in our laboratory’s previous research, resulting in the production of the F1 generation. The sterile line was chosen as the recurrent parent, and the fertile plants in the F1 generation and backcross progeny were selected for subsequent generations of backcrossing to produce a fertile near-isogenic line MR(KOTS)-90-110 (MR line). The cytoplasm of *Aegilops Kotschyi* serves as its cytoplasmic background. The thorough analysis of cytology and phenotypic reveals that, except from fertility and sterility, there are no differences between them in terms of other genetic backgrounds and agronomic features. This is excellent material for studying the sterility mechanism of K-type CMS wheat [34, 35]. However, the mechanism of K-type CMS wheat pollen abortion and its fertility regulation pathway are not clear enough.

In the present study, transcriptomics are used to analyze variations between a pair of near-isogenic lines of K-type CMS wheat MS(KOTS)-90-110 (MS line) and MR(KOTS)-90-110 (MR line) at different stages of anthers development, and a core transcriptional regulatory network in male sterile wheat was further constructed by bioinformatic analyses and various experimental verification. Meanwhile, these findings provide novel insights into the core mechanism of CMS in wheat and the pollen development process.

## Methods

### Plant materials and samples collection

In this study, a pair of near-isogenic lines of K-type CMS wheat—MS(KOTS)-90-110 (MS line) and MR(KOTS)-90-110 (MR line)—was used as the research material. The male sterile line with the *Ae. kotschyi cytoplasm* (KCMS, MS line) was crossed with the restorer line Rk5451, and then the MS line was used as the recurrent female parent to backcross with the F_1_ plants to construct a BC_7_F_1_ population. The fertile plants in BC_7_F_1_ population were considered to be its fertile near-isogenic MR line [36]. Seeds were sown in an experimental field of Northwest A&F University, Yangling, Shaanxi, China (108°82′E, 34°15′N) from 2017 to 2019 (as usual). 50 anthers were collected and mixed at each of the early uninucleate stage and binucleate stage of microspore development of MR line and MS line, respectively. All samples were immediately frozen in liquid nitrogen and stored at − 80 °C with three biological replications each sample for further analysis. Meanwhile, anthers obtained from both MS and MR lines in five stages (tetrad, early uninucleate, late uninucleate, binucleate and trinucleate stages) were stored in formalin-acetic acid-alcohol (FAA) and glutaraldehyde solution for phenotypic observations. All experiments were repeated three times.

### Phenotypic characterization and microspore analysis of wheat

Wheat anthers were observed under a Motic K400 dissecting microscope (Preiser Scientific, Louisville, KY, USA) and photographed using a Nikon E995 digital camera (Nikon, Tokyo, Japan). The stages of microspore development were identified by staining the anthers with 1% acetocarmine and 2% I_2_-KI. Samples were photographed using a DS-U2 high-resolution camera mounted on a Nikon ECLIPSE E600 fluorescence microscope (Nikon, ECLIPSE, E600, Tokyo, Japan) and analyzed using NIS-Elements software (Nikon, Tokyo, Japan) [37]. The fixed anthers were squashed in DAPI (40,6-diamidino-2-phenylindole) staining solution (0.1 M sodium phosphate, pH 7.0, 1.0 mM EDTA and 1 mg/ml DAPI) on microscope slides to observe the Chromosomes of microspore. Scanning electron microscopy (SEM) was used to characterize the surface characteristics of the pollen. Anthers fixed in 4% glutaraldehyde were treated with alcoholgradient, dried, and broken in sequence. Finally, the anthers and pollen grains were mounted on a stub with colloidal silver and photographed using a JSM-6360LV scanning electron microscope (JEOL, Tokyo, Japan) [38].

### Phytohormone (ABA and IAA) quantification

The anthers of MS line and MR line at the early uninucleate stage and binucleate stage of microspore were ground with liquid nitrogen respectively, and weigh about 0.2 g sample for quantitative determination of ABA and IAA phytohormones. Samples were collected and extracted using methanol compounds [39]. First, add 1mL precooled reagent I (methanol: water: acetic acid = 80:20:1) to each tube of sample, and extract it overnight at 4℃. The next day, 8000 g of the sample was centrifuged for 10 min, and the residue was extracted with 0.5mL reagent I (methanol: water: acetic acid = 80:20:1) for 2 h, and then centrifuged to take the supernatant. Blow all the supernatant obtained in the above steps with nitrogen at 40 ℃ until it contains no organic phase. Then, add 0.5mL reagent II (petroleum ether) to extract and decolorize for three times at 60℃-90℃ and discard the upper ether phase, then add reagent III (saturated citric acid aqueous solution) to adjust the PH to 2.8. Finally, use reagent IV (ethyl acetate) to extract the combined organic phase for three times, blow the nitrogen until it is dry, then add 0.5mL reagent V (methanol) to dissolve it by vortex vibration, and filter it for testing. RIGOL L3000 high performance liquid chromatograph (HPLC) is used to detect the content of endogenous ABA and Auxin in samples.

### RNA extraction and sequencing

Total RNA was extracted using RNAprep Pure Plant Kit (Tiangen, Beijing, China). RNA degradation and contamination was monitored on 1% agarose gels, and the RNA purity was checked using the NanoPhotometer® spectrophotometer (IMPLEN, CA, USA). RNA concentration was measured using Qubit® RNA Assay Kit in Qubit®2.0 Flurometer (Life Technologies, CA, USA), and RNA integrity was assessed using the RNA Nano 6000 Assay Kit of the Bioanalyzer 2100 system (Agilent Technologies, CA, USA). RNA quality testing is done by the cooperative company (Novogene, Beijing, China). A total amount of 3 µg RNA per sample was used as input material for the RNA sample preparations. Sequencing libraries were generated using NEBNext® UltraTM RNA Library Prep Kit for Illumina® (NEB, USA) and index codes were added to attribute sequences to each sample. First strand cDNA was synthesized using random hexamer primer and M-MuLV Reverse Transcriptase (RNase H^−^). Second strand cDNA synthesis was subsequently performed using DNA Polymerase I and RNase H. cDNA Library Preparation was performed as previously described [40]. At last, PCR products were purified (AMPure XP system) and library quality was assessed on the Agilent Bioanalyzer 2100 system. After cluster generation, the library preparations were sequenced on an Illumina Hiseq platform and 125 bp/150 bp paired-end reads were generated.

### Quality control and Identification of DEGs

Raw data (raw reads) of fastq format were firstly processed through in-house perl scripts. In this step, clean data (clean reads) were obtained by removing reads containing adapter, reads containing ploy-N and low quality reads from raw data. At the same time, Q20, Q30 and GC content the clean data were calculated. All the downstream analyses were based on the clean data with high quality. Reference genome and gene model annotation files were downloaded from genome website directly (IWGSC_RefSeq, ver. 1.0, Shengwei Ma, Nanjing, China, https://urgi.versailles.inra.fr/download/iwgsc/IWGSC_RefSeq_Assemblies/v1.0, accessed on 20 April 2020). Index of the reference genome was built using Hisat2 (v2.0.5) and paired-end clean reads were aligned to the reference genome using Hisat2 (v2.0.5). We selected Hisat2 as the mapping tool for that Hisat2 can generate a database of splice junctions based on the gene model annotation file and thus a better mapping result than other non-splice mapping tools [41]. The mapped reads of each sample were assembled by StringTie (v1.3.3b) [42]. The fragments per kilobase million (FPKM) were used to calculate the expression levels of genes. The differential gene expression analysis using the DEseq 2 R package (1.16.1) [43], with |log_2_(foldchange)|≥1 and FDR < 0.01. The *p* values obtained were adjusted by using Benjamini and Hochberg’s approach for controlling the FDR. Genes with an adjusted *p* value < 0.05 were finally assigned as DEGs.

### Functional annotations of DEGs and construction of the putative network model

GO annotation and KEGG pathway analyses were performed for the DEGs. Gene Ontology (GO) enrichment analysis of differentially expressed genes was implemented by the clusterProfiler R package, in which gene length bias wascorrected [44]. GO terms with corrected *p*-value less than 0.05 were considered significantly enriched by differential expressed genes. Then clusterProfiler R package was used to test the statistical enrichment of differential expression genes in KEGG (Kyoto Encyclopedia of Genes and Genomes) pathways [45]. KEGG enrichment analysis with a corrected *p*-value ≤ 0.05 were considered to be significantly enriched The heatmap was drawn using TBtools software [46]. According to the log_2_(foldchange) of DEGs between MS line and MR line, DEGs are annotated and classified by MapMan 3.6.0RC1 (https://mapman.gabipd.org/web/guest/ mapman-version-3.6.0 ) software [47, 48]. We used the software of Adobe Illustrator CS5 (Adobe, CA, USA) and Science Slides Suite to draw the network model.

### qRT-PCR analysis for gene expression

The relative expression levels of selected genes were determined using TB Green®Premix Ex Taq™ (TaKaRa, Shiga, Japan) after cDNA was synthesized. The qRT-PCR reaction was performed on a 10 µL scale using SYBR Premix Ex Taq™ (TaKaRa, Shiga, Japan) on an Eco Real-Time PCR System (Illumina, CA, USA). The relative expression levels were calculated by the 2^−∆∆Ct^ method [49]. At least three biological replicates and three technical replicates were performed for each sample. The Actin gene (GenBank accession: 542,814) in wheat was used as a reference gene. The Integrated DNA Technologies website (https://sg.idtdna.com/scitools/Applications/RealTimePCR/Default.aspx, accessed on 20 September 2020) was used for the design of primers targeting gene-specific regions and then listed in Table S5 (Table S5).

## Results

### Characterization of anther and microspore development of the CMS Line MS and the near-isogenic lines MR

**Fig. 1:**
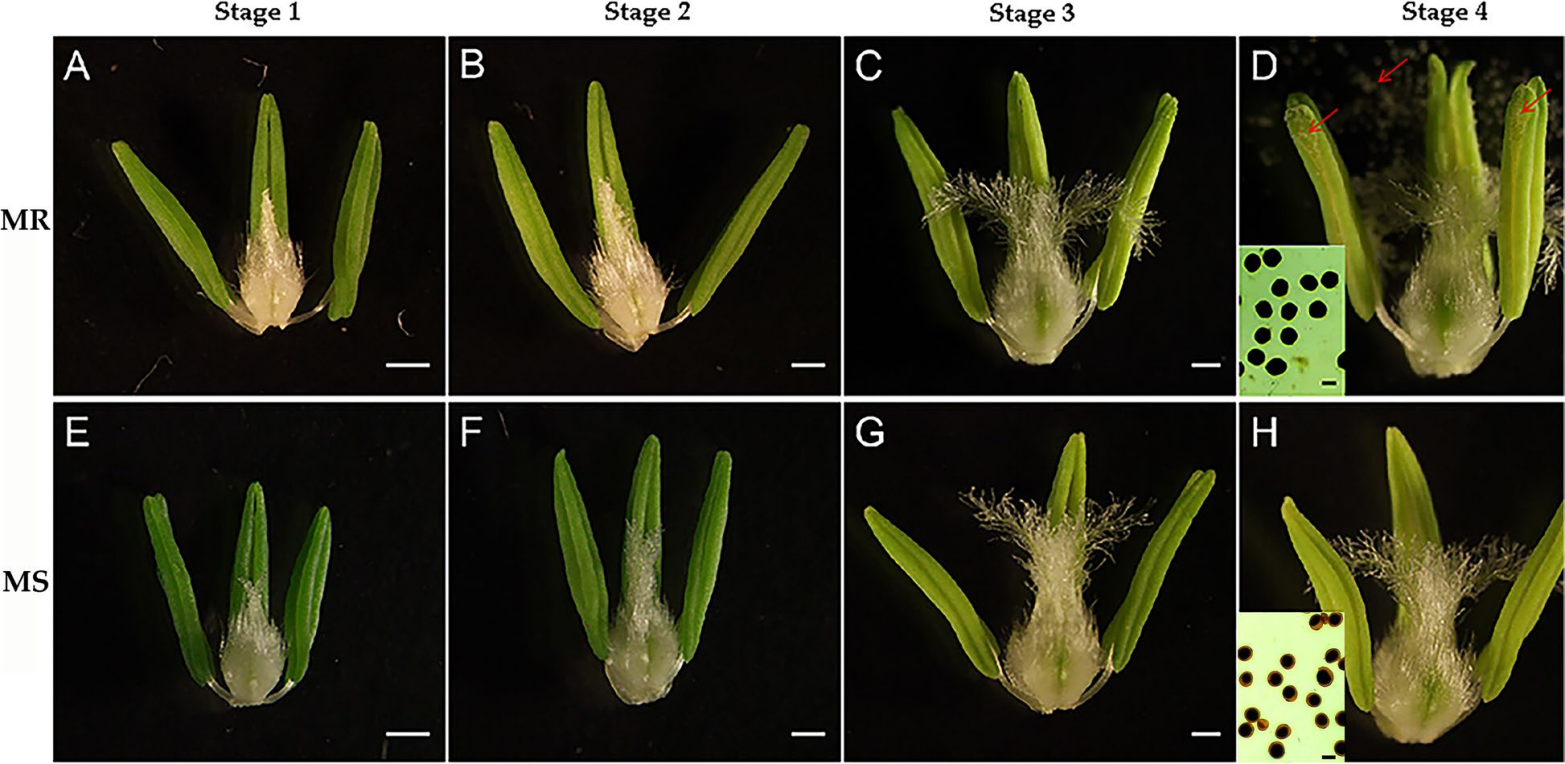
Phenotypes of anthers and pistils in the male-sterile (MS) line and its near-isogenic male fertile (MR) line. stage 1: early uninucleate stage (**A, E**); stage 2: late uninucleate stage (**B, F**); stage 3:binucleate stage(**C, G**); stage 4:trinucleate stage (**D, H**). the 2% I2-KI staining pollen grains (**D**, **H**, bottom left). Red arrows indicate mature pollen grains (**D**). Scale bars: 0.5 mm in (**A- H**), 50 µm in the bottom left of(**D, H**)

 Based on morphological characteristic or cellular events visible under the light microscope and previous study of classification of anther development [50, 51], we divided wheat anther development into four stages. First of all, MS line and MR line plant pistils showed normal development (Fig. 1). However, from the early-uninucleate stage to the trinucleate stage, the anthers of MS line were smaller than those of MR line (Fig. 1A-D, E-H). More importantly, at the trinucleate stage, unlike MR line anthers, the anthers of MS line could abnormally dehisce and unable to release pollen grains (Fig. 1D, H). Moreover, Iodine-potassium iodide (I2- KI) staining showed that the mature pollen grains of the MR line were full, regular, and dyed black (Fig. 1D, bottom left); however, the pollen cells of the MS line were wrinkled, irregular, and the staining was a brown and uneven (Fig. 1H, bottom left).

**Fig. 2:**
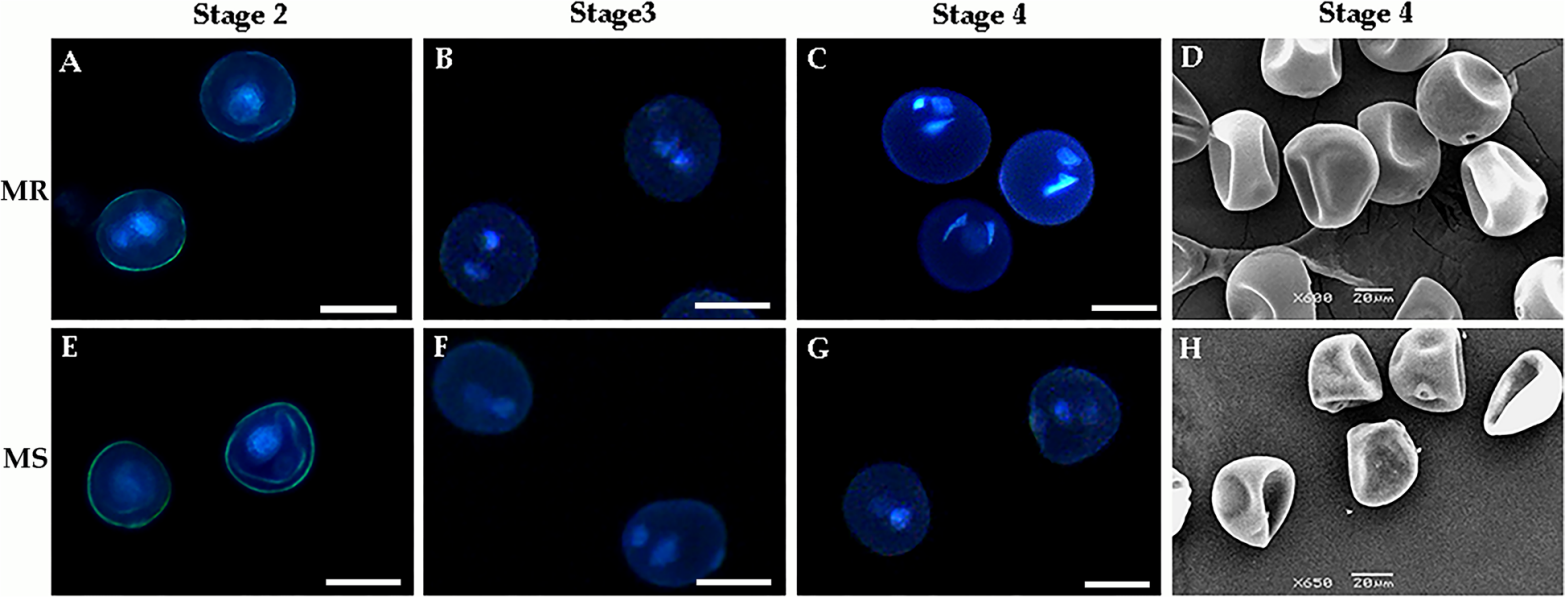
Cytological and morphological observations of microspores in the male-sterile (MS) line and its near-isogenic male fertile (MR) line. stage 2: uninucleate stage (**A, E**); stage 3:binucleate stage (**B, F**); stage 4:trinucleate stage (**C, G**). Scale bars: 50 µm in (**A - G**),20 µm in (**D, H**)

 In order to identify the stages of abnormal pollen development, we carried out cytological observations on developing microspores from the MS and MR lines. DAPI staining showed that there was no significantly difference between MS and MR line at uninucleate stage (Fig. 2A, E). At the binucleate stage, the microspores of MR line at binucleate stage contained one intensely stained sperm nucleus and one dispersed, weakly stained vegetative nucleus (Fig. 2B); however, there are two dispersed, weakly stained vegetative nucleus in the microspores of MS line (Fig. 2F). More importantly, at the trinucleate stage, the microspores of MR line were turgid and round, and the nucleus divided normally into two sperm nucleus and one vegetative nucleus (Fig. 2C). By contrast, the microspores of MS line were irregular in shape, plasmolysis occurred, and the nucleus failed to divide normally into two sperm nucleus (Fig. 2G).

Scanning electron microscopy (SEM) further showed that the pollen grains of the MS line showed more obviously abnormal, sunken morphology at the trinucleate stage than those of MR line (Fig. 2D, H). These results indicated that the abnormal microspore development of MS line was mainly manifested in the binucleate stage of anther development.

#### Overview of RNA-Seq data analysis

In order to investigate the molecular mechanism in abnormal anther and pollen development of MS(KOTS)-90-110, transcriptomics analysis was performed by RNA-Seq using anther samples in early uninucleate stage and binucleate stage under fertile plants (named MRu and MRb for each developmental stage, respectively), and sterile plants (named MSu and MSb for each developmental stage, respectively). Each anther sample contains three individual biological replicates. In total, 12 libraries (MSu-1, MSu-2, MSu-3, MSb-1, MSb-2, MSb-3, MRu-1, MRu-2, MRu-3, MRb-1, MRb-2, MRb-3) were sequenced and 696,085,410 clean reads were obtained, with 343,418,502 reads from MS line and 352,666,908 from MR line. The percentage of Q20 exceeded 94.88%, and the GC content for the clean data ranged from 49.93 to 52.41%. The alignment efficiency of clean reads from each sample ranged from 79.78 to 93.72% compared with the wheat reference genome (Table 1). FPKM violin analysis detected no bias in the construction of the cDNA libraries and the gene expression levels in each sample (Fig. S1). Correlation heat map analysis detected high correlations between the biological replicates (Fig. S2). The results demonstrated that the quality of the transcriptome sequencing was reliable and sufficient for further analysis.

**Table 1:** Summary and evaluation of transcriptome-sequencing data

Sample ID	Total reads	Clean reads	GC Content(%)	Q20(%)	Mapped reads(%)
MSu1	54,731,562	52,414,108	49.93	97.31	83.36
MSu2	63,244,572	61,740,392	50.8	97.7	88.31
MSu3	48,430,392	47,117,986	50.77	97.13	85.73
MSb1	49,200,240	46,686,688	51.86	97.65	85.96
MSb2	60,465,716	58,748,790	52.41	97.56	79.78
MSb3	79,164,226	76,710,538	50.78	94.88	93.72
MRu1	48,435,474	46,906,350	50.15	97.39	85.07
MRu2	48,981,082	47,357,918	50.69	97.54	84.22
MRu3	65,629,362	61,460,370	51.54	97.48	84.37
MRb1	62,271,834	59,953,598	51.61	97.58	90.17
MRb2	54,772,202	53,058,046	52.14	97.7	87.36
MRb3	86,473,152	83,930,626	50.74	95.09	93.54

#### Screening of differentially expressed genes

**Fig. 3:**
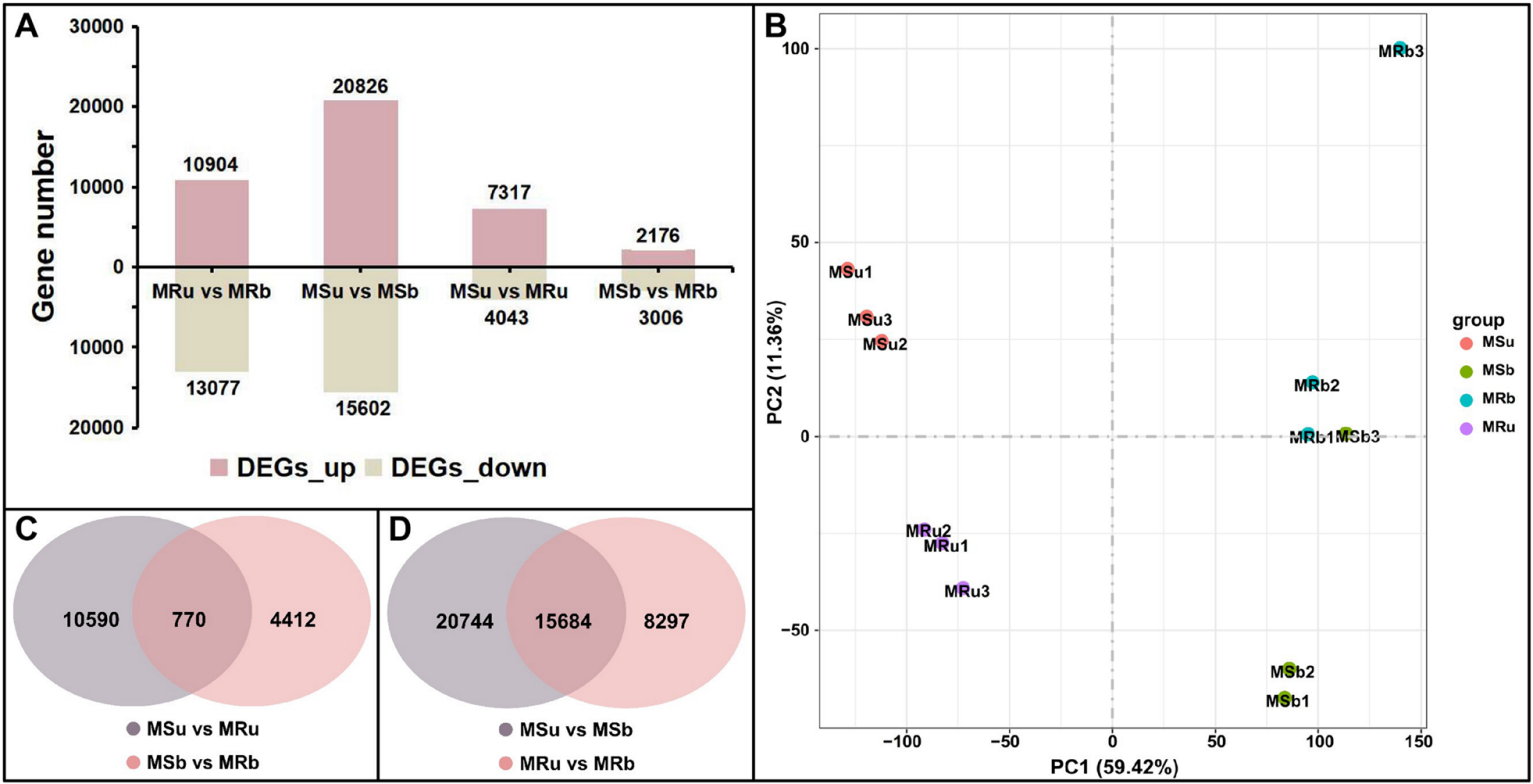
Analysis of DEGs in the male-sterile (MS) line and its near-isogenic male fertile (MR) line. **A **Number of differentially expressed genes (DEG) detected in different fertility materials and different development stages. **B **PCA clustering based on transcriptome data. **C** Venn diagrams of DEGs in MSu vs MRu and MSb vs MRb. **D **Venn diagrams of DEGs in MSu vs MSb and MRu vs MRb

Using RNA-seq, differentially expressed genes were detected in the sterile and corresponding near-isogenic lines. Differentially expressed genes (DEGs) were detected based on screening criteria (|log_2_(foldchange)| ≥1, *p*-value < 0.05) in MS line and MR line (MSu vs. MRu, MSb vs. MRb, MSu vs. MSb, MRu vs. MRb). After computing the expression values, 11,360, 5,182, 36,428 and 23,981 DEGs exhibited different expressions between MSu vs. MRu, MSb vs. MRb, MSu vs. MSb and MRu vs. MRb libraries, respectively (Table S1). There were 11,360 DEGs detected between MSu vs. MRu libraries, of which 4,043 were downregulated and 7,317 were upregulated. A total of 5,182 DEGs in MSb vs. MRb libraries, among which 3,006 were downregulated and 2,176 were upregulated. For 36,428 DEGs identified in MSu vs. MSb libraries, 15,602 were downregulated and 20,826 were upregulated. Of the 23,981 DEGs in MRu vs. MRb libraries, 13,077 were downregulated and 10,904 were upregulated (Fig. 3A, Fig. S3).

Based on gene expression, principal component analysis (PCA) was used to estimate the distance relationship among the 12 samples (Fig. 3B). PCA score plots showed a clear separation between samples from analyzed anther-developmental stages, and they were separated along PC1 with a left-to-right trend following the developmental stages (early uninucleate stage to binucleate stage); there was a significant difference between MSu and MRu, MSb and MRb, suggesting that the overall transcriptomic profiles between the MS line and MR line were distinct. For the DEGs between two plant materials with different fertility at the same development stage of anthers, 10,590 were specifically expressed at the early uninucleate stage, 4,412 at the binucleate stage, and 770 unigenes were co-differentially expressed (Fig. 3C). In addition, for the DEGs in anthers of different development stages with the same fertility, 20,744 were specifically expressed at the MS line, 8,297 at the MR line, and 15,684 unigenes were co-differentially expressed (Fig. 3D). This may suggest that at the early uninucleate stage and binucleate stages of anther development, the DEGs contain critical information related to male sterility.

#### Gene ontology annotation and pathway enrichment analysis of DEGs

**Fig. 4:**
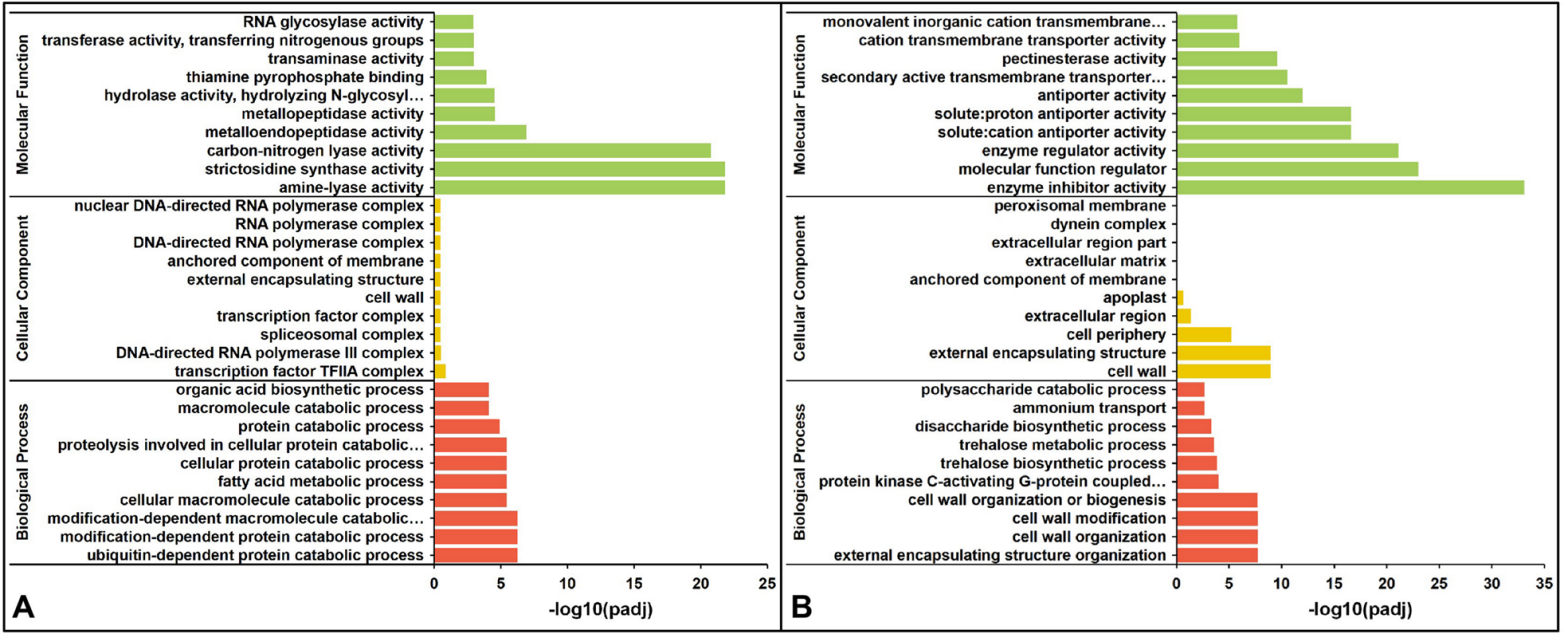
GO function enrichment of DEGs between the MS line and the MR line. Gene ontology (GO) analysis of DEGs in (**A**) MSu vs MRu and (**B**) MSb vs MRb.The top 20 enriched GO terms ranked by *p*-values are shown. MS: male sterile line; MR: near-isogenic male fertile line; u: early uninucleate stage; b: binucleate stage

 In order to explore the molecular mechanism of fertility difference between MS line and MR line, we carried out GO enrichment analysis and KEGG enrichment analysis on DEGs between fertile and sterile anthers at the early uninucleate stage and binucleate stages respectively. At the early uninucleate stage, a total of 66 GO terms enriched pathways were significantly enriched for DEGs at the early uninucleate stage between MS line and MR line (Table S2). At the early uninucleate stage, the top 30 terms were mostly enriched for amine-lyase activity, strictosidine synthase activity, carbon-nitrogen lyase activity, ubiquitin-dependent protein catabolic process, modification-dependent protein catabolic process and modification-dependent macromolecule catabolic process (Fig. 4A and Table S2). At the binucleate stage, the top 30 terms were mostly enriched for enzyme inhibitor activity, molecular function regulator, enzyme regulator activity, solute: cation antiporter activity, solute: proton antiporter activity, cell wall, external encapsulating structure, cell wall organization and modification or biogenesis (Fig. 4B and Table S2).

**Fig. 5:**
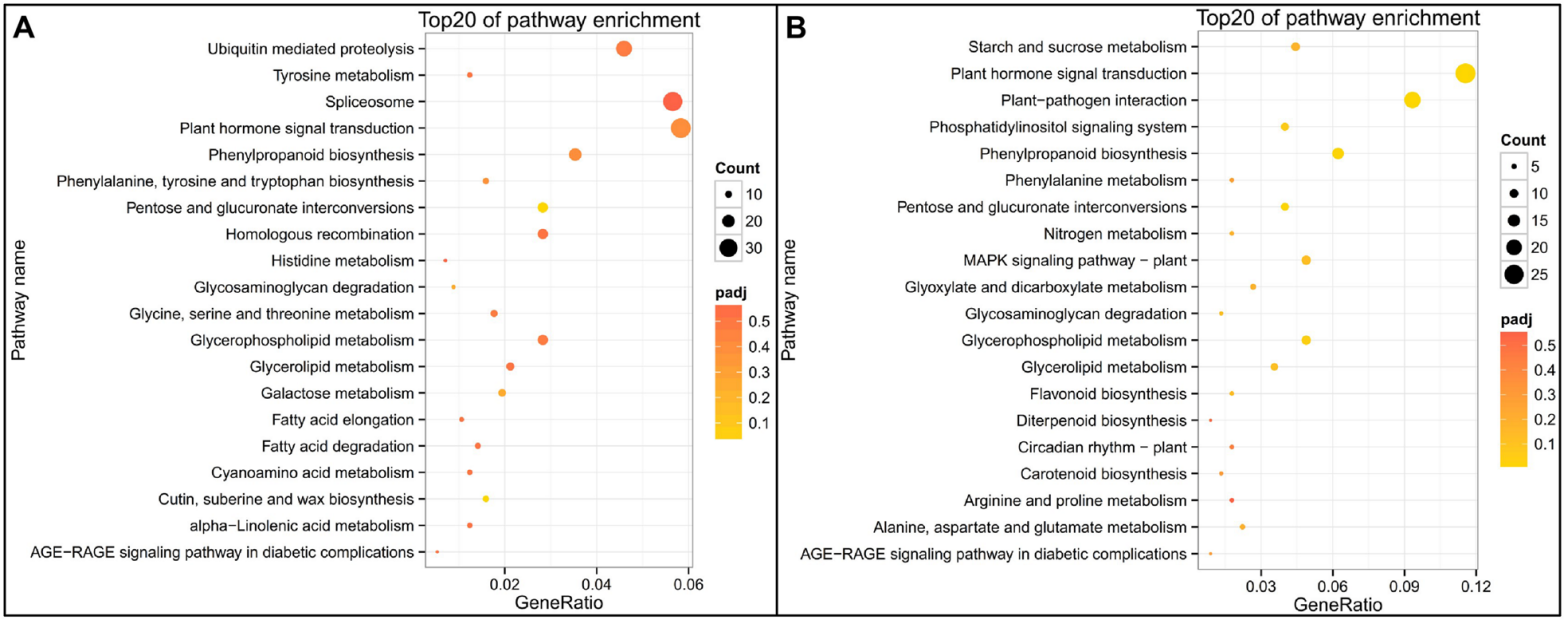
The top 20 significantly enriched pathwaysbased on KEGG between the MS line and the MR line. KEGG analysis of DEGs in (**A**) MSu vs MRu and (**B**) MSb vs MRb. MS: male sterile line; MR: near-isogenic male fertile line; u: early uninucleate stage; b: binucleate stage

In order to identify the functions of DEGs between MS line and MR line, the Kyoto Encyclopedia of Genes and Genomes (KEGG) pathway enrichment was used to identify major biochemical and signal transduction pathways where the DEGs participated [52–54]. At the early uninucleate stage, the top 20 pathways of the KEGG were mainly enriched in plant hormone signal transduction, spliceosome and phenylpropanoid biosynthesis (Fig. 5A, Table S3). Furthermore, at the binucleate stage, the top 20 terms mostly participated in plant hormone signal transduction, phenylpropanoid biosynthesis, lipid metabolism, MAPK signaling pathway to the plant, flavonoid biosynthesis, and starch and sucrose metabolism (Fig. 5B, Table S3). These results suggested that multiple complex metabolic pathways were involved in the anther development of CMS wheat.

### Expression patterns of DEGs of the plant hormone signal transduction pathway, endogenous hormone measurements and validation of related-DEGs by qRT-PCR

**Fig. 6:**
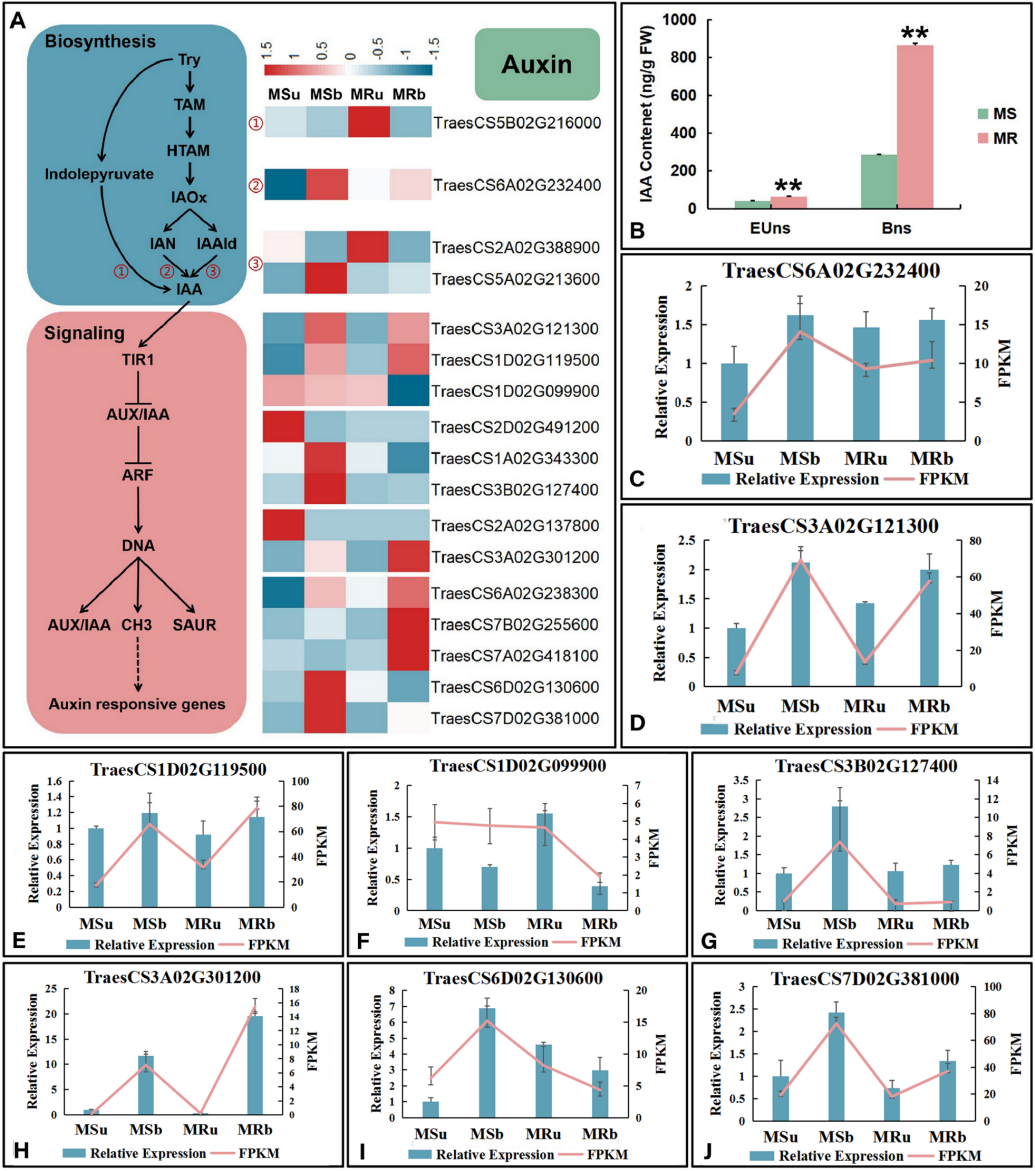
Expression levels of DEGs of the auxin metabolism pathway. **A **Expression patterns of DEGs of the IAA biosynthesis and signaling pathway. **B **the contents of IAA. The significance of differences was assessed using the Student’s t-test. *p < 0.05, ** *p* < 0.01.** C-J** qRT-PCR analysis of the expression patterns of randomly selected IAA-related DEGs. Data are presented as the means ± SD of three technical replicates and three independent biological replicates

**Fig. 7:**
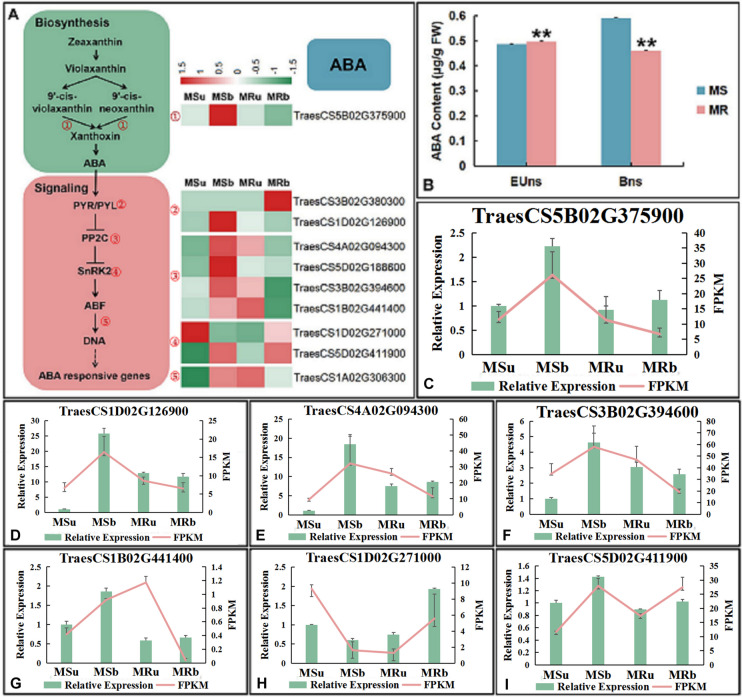
Expression levels of DEGs of the abscisic acid metabolism pathway. **A** Expression patterns of DEGs of the ABA biosynthesis and signaling pathway. **B** the contents of ABA. The significance of differences was assessed using the Student’s t-test. *p < 0.05, ** *p* < 0.01. **C**-**J** qRT-PCR analysis of the expression patterns of randomly selected ABA-related DEGs. Data are presented as the means ± SD of three technical replicates and three independent biological replicates

The results of KEGG analysis showed that DEGs involved in plant hormone signal transduction pathway were predominantly enriched during the anther development of wheat (Fig. 5). In order to understand the hormonal regulation of abnormal anther fertility in more detail, the expression levels of pivotal DEGs in the auxin (IAA) (Fig. 6) and abscisic acid (ABA) (Fig. 7) signaling pathways were analyzed.

Four DEGs were identified in the IAA biosynthetic pathway. *TraesCS5B02G216000* and *TraesCS2A02G388900* are mainly expressed in the early uninucleate stage of MR line, but *TraesCS6A02G232400* and *TraesCS5A02G213600* are mainly expressed in the binucleate stage of MS line. In addition, large amounts of DEGs are enriched into various parts of the IAA signal transduction pathway. For example, at the early uninucleate stage, *TraesCS2D02G491200* (auxin response factor, ARF) and *TraesCS2A02G137800* (auxin-responsive, GH3) were upregulated in MS line. Meanwhile, *TraesCS3A02G121300, TraesCS1D02G119500* (AUX/ IAA-related genes) and TraesCS6A02G238300 (auxin-responsive gene, SAUR) were downregulated in MS line. Moreover, at the binucleate stage, *TraesCS3B02G127400*, *TraesCS1A02G343300* (AUX/ IAA-related genes) were upregulated in MS line but TraesCS7A02G418100, *TraesCS7B02G255600* (auxin-responsive gene, SAUR) were downregulated (Fig. 6A).

In the ABA biosynthetic pathway, *TraesCS5B02G375900* (9-cis epoxycarotenoid dioxygenases, NCED) is mainly expressed in the binucleate stage of MS line. For ABA signal transduction pathway, DEGs enriched in PP2C phosphatase process are downregulated in the early uninucleate stage of MS line, but upegulated in the binucleate stage; *TraesCS1D02G126900* (ABA receptor, PYR/PYL) is mainly expressed in the binucleate stage of MS line. In addition, Snf1-related protein kinase related genes are also differentially expressed at different levels in MSline and MR line (Fig. 7A). The results suggested that plant hormone signal transduction pathway play an important role in regulating the development of anther and microspore in wheat.

We measured the contents of endogenous hormones, including IAA and ABA of MS line and MR line during the two stage of anther development. IAA contents in both sterile and fertile plants was lower in the early uninucleate stage than in the binucleate stage, and it was always lower in MS line than in MR line (Fig. 6B). Meanwhile, ABA contents in MS line were lower than those in MR line at the early uninucleate stage, but the ABA content in MS line was significantly higher than that in MR line at the binucleate stage (Fig. 7B). Anther samples were collected from the MS line and MR line and the expression levels of some key DEGs of the hormone biosynthesis and signaling pathway were measured by qRT-PCR. The expression pattern of eight genes of the IAA and seven genes of the ABA pathways were analyzed by qRT-PCR (Figs. 6C and 7C-I). The expression tendencies were consistent with the RNA-Seq results, indicating that the transcriptome sequencing results were accurate and reliable in this study.

### Expression patterns of the related-DEGs of MAPK signal transduction and spliceosome between the MS line and the MR line

**Fig. 8:**
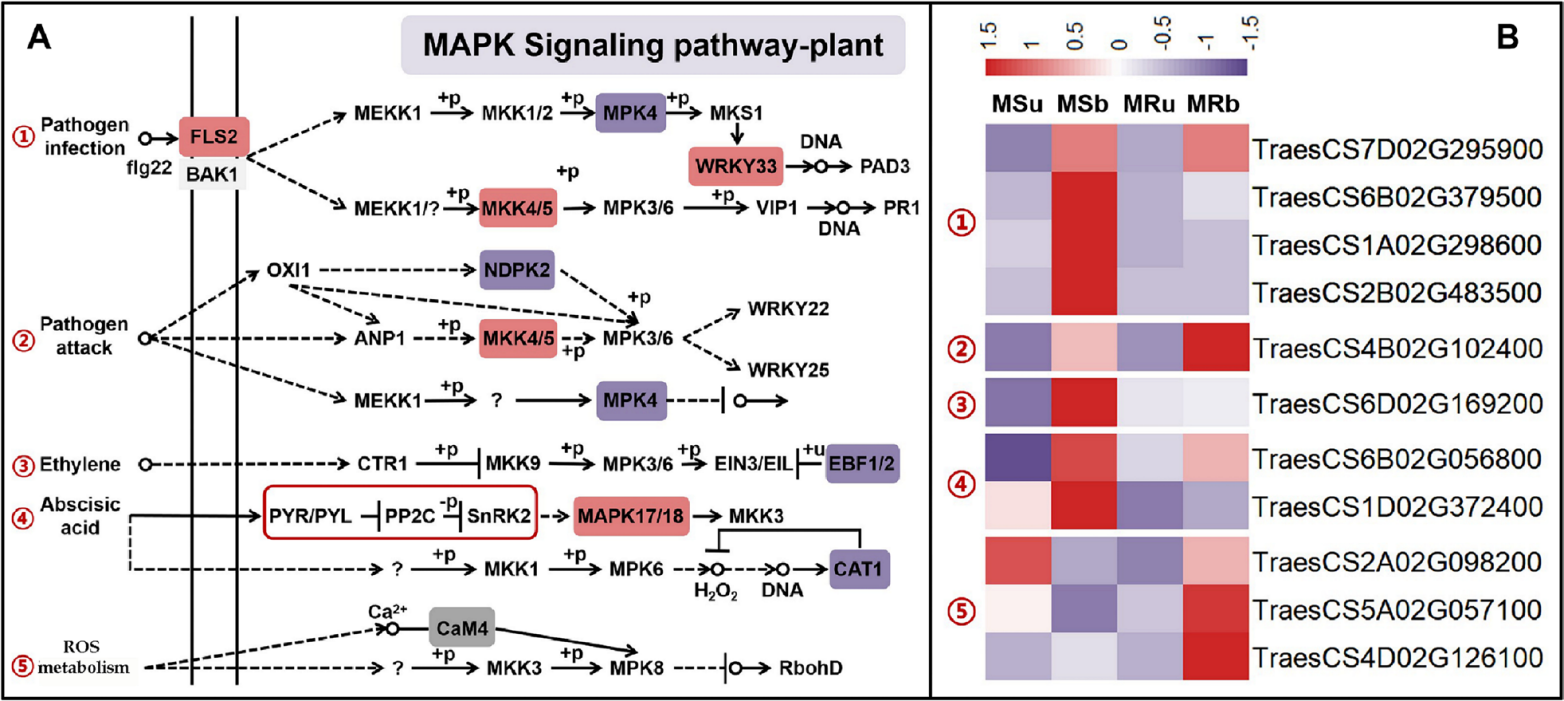
The expression levels of differentially expressed genes involved in MAPK signal transduction. **A** Schematic of MAPK signal transduction in the anther of wheat. **B **Heatmap representation of differentially expressed genes involved in the MAPK signal transduction in MS line and MR line

**Fig. 9:**
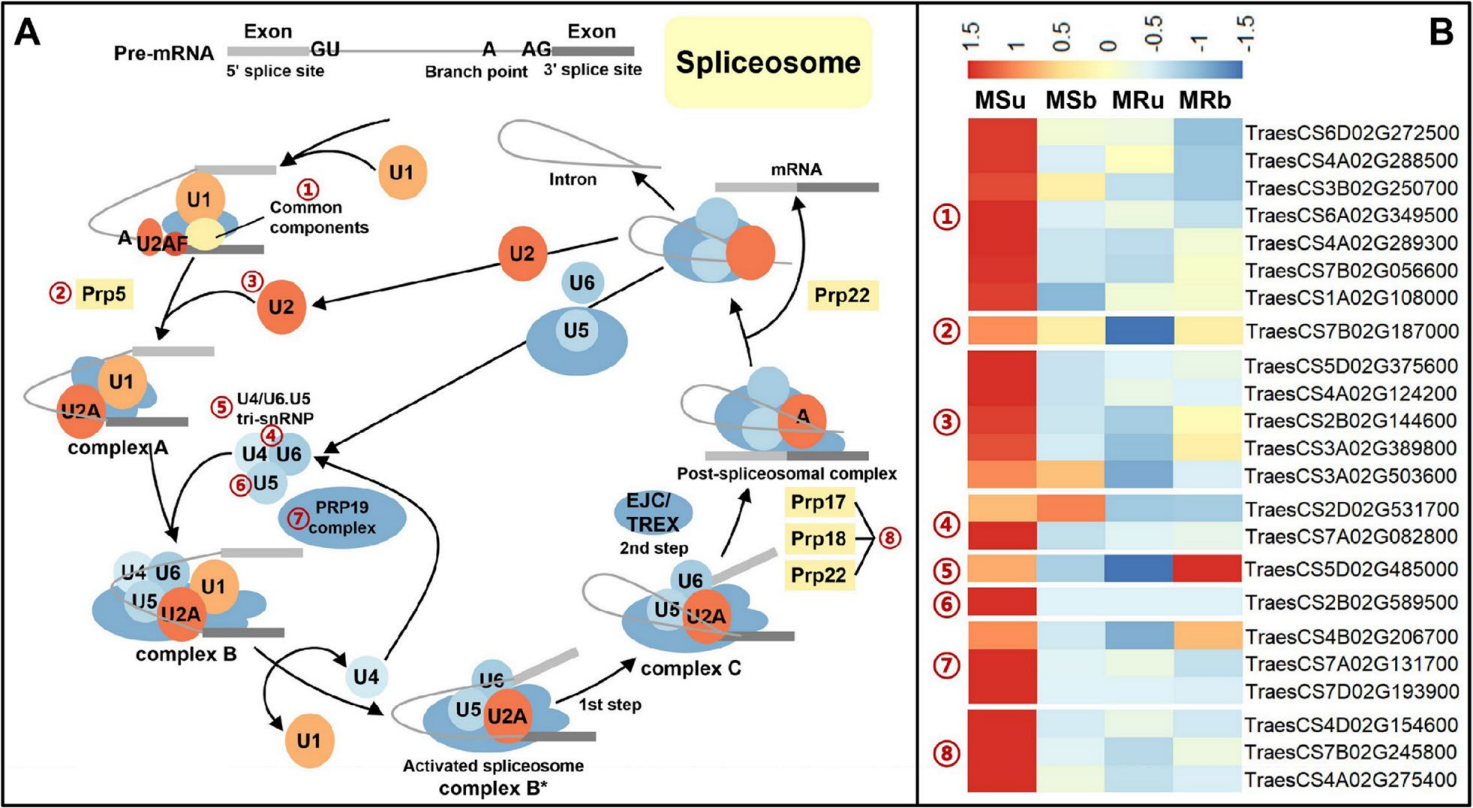
The expression levels of differentially expressed genes involved in alternative splicing. **A** The schematic diagram of alternative splicing and the structure of spliceosome. **B** Heatmap representation of differentially expressed genes involved in spliceosome in MS line and MR line

MAPKs (mitogen-activated protein) belong to Ser/Thr protein kinases, which are ubiquitous and highly conserved in eukaryotes and are mainly responsible for signal transduction and amplification from extracellular to intracellular [55]. The MAPK signal pathways are not only involved in plant biological and abiotic stress, plant hormone signal transduction, but also in many plant growth and development processes, including reproductive growth and development. In this transcriptional analysis, we identified 11 mitogen-activated protein kinase (MAPK) signal transduction-related genes between MS line and MR line (Fig. 8). Among them, 5 DEGs participating in plant-pathogen interaction are mainly expressed in the binucleate stage, and they were mainly upregulated in the binucleate stage of MS line. In addition, MAPK related DEGs in our plant materials also participate in plant hormone transduction pathway. *TraesCS6D02G169200* participates in ethylene (ET) regulation through the MAPK cascade pathway of CTR1/MAP3K-MKK9-MPK3/6-EIN3 mode [56], *TraesCS6B02G056800* and *TraesCS1D02G372400* are involved in abscisic acid signal transduction pathway, and these three DEGs were upregulated in the binucleate stage of MS line. Moreover, three DEGs were identified as participating in ROS metabolism pathway mediated by MAPK cascade reaction (Fig. 8A,B). Alternative splicing is widely found in plants and is the main source of plant transcriptome and proteome diversity [57, 58]. Alternative splicing is completed by spliceosome, which is a 40-60 S ribonucleoprotein complex [59]. The results of KEGG enrichment showed that a large amount of DEGs was enriched in the structure of spliceosome at the early uninucleate stage (Fig. 5). Here, we analyzed the expression level of 23 DEGs related to spliceosome. Seven DEGs participate in the composition of the Common component, and their main function is to identify pre-mRNA; *TraesCS4B02G206700*, *TraesCS7A02G131700* and *TraesCS7D02G193900* in seven PRP (Precursor RNA Processin) related DEGs participate in the formation of PRP19 complex; 5 DEGs related to U2 snRNPs (small nuclear ribonucleoproteins) function, and 4 DEGs related to the formation process of U4/U5/U6 complex (Fig. 9). More importantly, except that *TraesCS5D02G485000* is significantly up-regulated in the binucleate stage anthers of MR line, the expression level of other DEGs was higher in the early uninucleate stage anthers of MS line. These results indicated that MAPK signal transduction and AS are important for the normal development of CMS wheat anthers and microspores. At the same time, it is emphasized that there is a complex regulatory network in the process of wheat anther development.

#### MapMan pathway analysis of DEGs

**Fig. 10:**
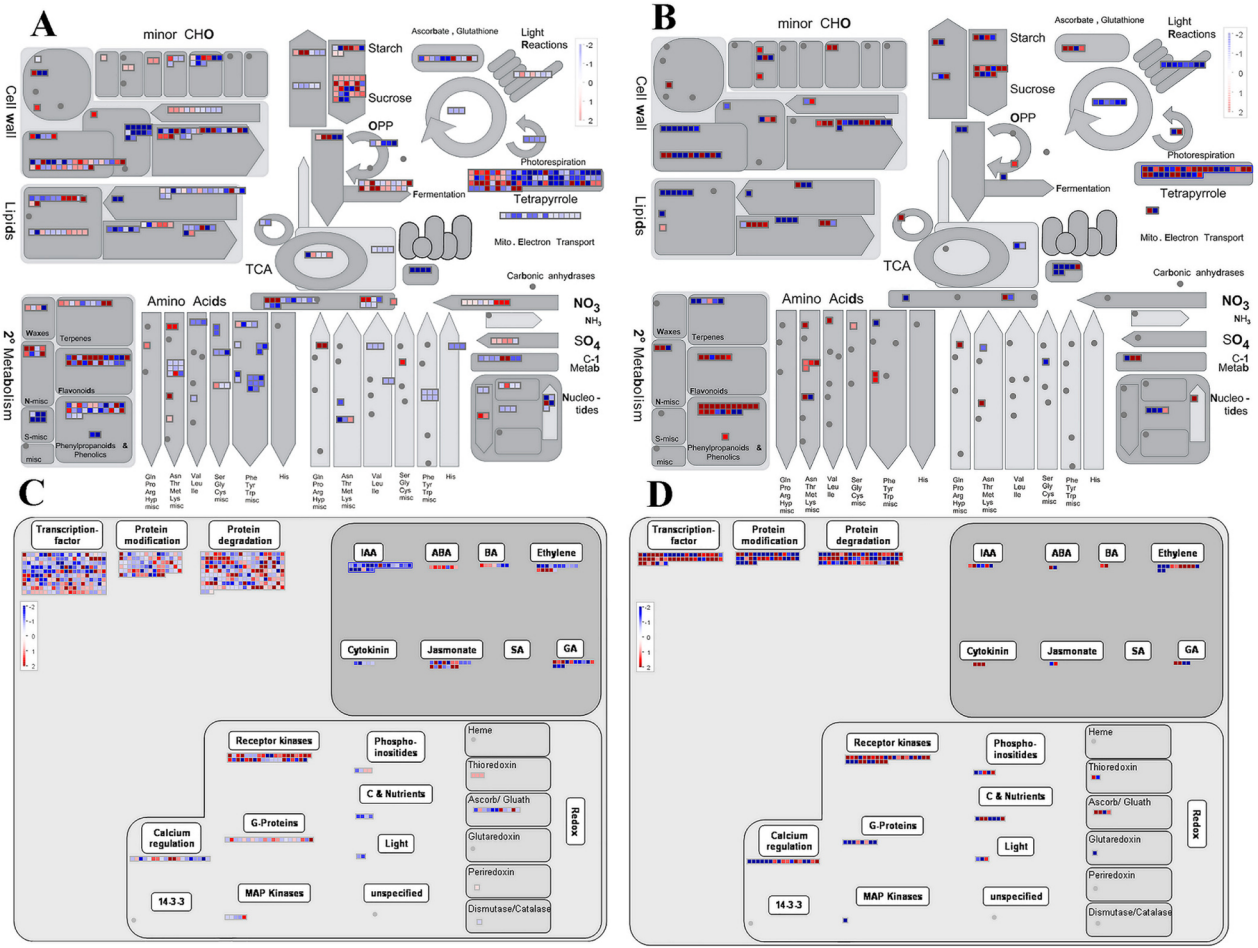
Functional classifcation of the DEGs of the anthers between MS line and MR line via MapMan analysis. **A, B** Metabolism overview. **C, D **Regulation overview. **A, C **MSu vs MRu and (**B, D**) MSb vs MRb. Each square represents a single differentially expressed gene. The log2 (foldchange) (log2FC) was loaded into MAPMAN to generate the color scale varying from -2 to 2. Dark red color indicates higher expression in MS line compared with MR line, and dark blue color signifies more expression in MR line compared with MS line

In order to comprehensively and intuitively understand the enrichment of differentially expressed genes and the pathways involved in them, we have made MapMan annotation on the DEGs identified between MS line and MR line at the early uninucleate and binucleate stages, respectively. The wheat-specific Taes_AFFY_0709 mapping file (Table S6) was used as the background file for DEGs annotations (Table S4). The results showed that in the early stage of microspore development, DEGs between fertile and sterile plants were mainly enrichment in plant hormone metabolism and signal transduction pathway, starch and sucrose metabolism pathway, cell wall formation and degradation processes, lipids metabolism pathway, TCA cycle processes and secondary metabolism pathway (Fig. 10A). The expression level of ABA regulation processes DEGs in the early uninucleate stage of MS line was higher than that of MR line, but the expression level of IAA regulation processes DEGs at this stage was in the opposite state (Fig. 10C). At the same time, the expression level of flavonoid, phenylpropanoids and lipids related DEGs also showed extremely significant differences in the anthers of two plant materials with different fertility. In the binucleate stage of microspore development, plant hormones, cell wall synthesis and degradation, lipid metabolism, starch and sucrose metabolism, TCA cycle-related enzymes, flavonoids and phenylpropanoids metabolism are still the main enrichment modules of DEGs. However, the expression level of these DEGs has changed in different plant material anthers. For example, the expression of DEGs related to ethylene synthesis and degradation was significantly up-regulated in MS line, and the expression of DEGs related to ethylene response pathway was significantly down-regulated. More importantly, the expression of DEGs related to phenylpropanoids metabolism in the anthers of MS line at the binucleate stage was significantly higher than that of MR line, which was contrary to the results of the early uninucleate stage (Fig. 10B, D). These results further illustrated that the accuracy of the above findings, and set the direction for us to further explore the regulation processes of CMS wheat pollen development.

## Discussion

Wang et al. (2016) sequenced and analyzed the anthers of cytoplasmic male sterile lines and corresponding maintainers of Chinese cabbage through RNAseq technology. The results showed that a total of 5592 differentially expressed genes were identified in sterile lines and fertile lines, and these differentially expressed genes were mainly enriched in energy metabolism, carbon water compound synthesis, heat shock protein synthesis, coding transcription factors and other metabolic pathways [60]. The anthers of K-TCMS male sterile line were analyzed by transcriptome sequencing technology, and a total of 16,840 differentially expressed genes, mainly involving jasmonic acid biosynthesis, MYB transcription factor transcriptional regulation and phenylpropanol biosynthesis [61]. The anthers of wheat physiological male sterility induced by SQ-1 at five development stages were analyzed by RNAseq. The results showed that most genes were involved in plant hormone signal transduction, phenylpropanoid biosynthesis, starch and sucrose metabolism, amino acid biosynthesis, etc. In the present study, we used transcriptome sequencing to analyze the dynamic profiles of gene expression between a pair of near-isogenic lines of K-type CMS wheat-MS(KOTS)-90-110 (MS line) and MR(KOTS)-90-110 (MR line) at different stages of anthers development. The results showed that the DEGs between MS line and MR line mainly connects with plant hormone, MAPK signal transduction and the composition of spliceosome, and so on. Thus, the regulation mechanism of CMS wheat pollen fertility is a complex regulatory network at the transcriptome level. The results vary from material to material, and may also vary depending on the fertility gene locus.

### IAA and ABA signal transmission pathway participates in wheat pollen development

In recent years, with the isolation and functional identification of many genes related to rice anther development, many plant hormone related genes have been found to play an important role in rice anther development [62, 63]. Studies have shown that the dynamic balance of plant hormones such as auxin, abscisic acid, gibberellin, jasmonic acid and cytokinin will affect the vegetative and reproductive growth of plants [64, 65]. Among them, a large number of studies have revealed the important role of plant hormones in regulating the development of anther [66]. Under high temperature, the level of endogenous auxin in developing anthers of barley and *Arabidopsis* decreased, leading to abortion of pollen development, but this phenomenon can be recovered by spraying auxin externally [19]. At each stage of anther development, excessive or insufficient abscisic acid content may lead to male sterility [67]. Therefore, the optimal abscisic acid level at different development stages is essential for normal anther development.

A large amount of evidence shows that auxin plays its role by controlling its synthesis, transportation and signal transduction [68, 69]. At present, research on the synthesis pathway of auxin mainly focuses on the tryptophan dependent pathway. According to the different main intermediate products in the IAA synthesis process, it is usually divided into four branches, namely IPA (indole-3 -pyruvic acid), IAOx (indole-3-acetate), TAM (tryptamine), and IAM (indole-3-acetate) pathways [70]. The complex network of auxin synthesis pathways leads to complex regulatory patterns in this process. Therefore, more research is needed to explain the correlation between the levels of endogenous hormone content in anthers and the expression levels of related genes. Nitrilase (NIT) is widely present in plants, and its main function is to hydrolyze organic cyanide into ammonia and the corresponding carboxylic acids [71]. Cyanides, which are by-products of plant ethylene synthesis, are mostly toxic. Therefore, NIT can play a detoxifying role [72]. In *Arabidopsis*, the NIT family consists of four members (*NIT1*, *NIT2*, *NIT3*, and *NIT4*), among which *NIT1-3* are collectively referred to as the AtNIT1 subfamily. In addition to their detoxification effects, they also participate in the biosynthesis of IAA. However, *NIT4* has a strong substrate specificity, and its main function is to participate in the detoxification process of toxic cyanide in plants [73]. In addition, most *NITs* in the plant kingdom are homologous to Arabidopsis *NIT4*, such as *NIT1* and *NIT2* in maize. Interestingly, NIT2 can hydrolyze IAN and participate in the biosynthesis of IAA [74]. We conducted evolutionary and protein structure analyses on *TraesCS6A02G232400* and found that, although it belongs to the NIT family, it may function as a *NIT4* gene (Fig. 6A). It is worth noting that we have found that DEGs related to the ethylene regulatory pathway are also enriched to some extent. Therefore, *TraesCS6A02G232400* remains one of the candidate genes that are worth further functional verification. When plants encounter stress, they generally produce reactive oxygen species (ROS), which can often impede the normal growth of plant cells [75]. Excessive aldehydes in plants can lead to metabolic disorders [76]. Acetaldehyde dehydrogenase (ALDHs) can maintain the normal physiological function of plant cells by degrading harmful aldehydes in the plant and clearing ROS [77]. On the other hand, ALDH also plays an important role in plant hormone metabolism, such as ABA, IAA, and GA (gibberellin) [78–80]. The latest research has found that the expression levels of two ALDH genes in sterile mango plants are higher than those in fertile plants [81]. In our study, *TraesCS5A02G213600* is likely to encode the ALDH protein (Fig. 6A). Therefore, it will also be investigated as a significant candidate gene in future research. In the auxin signal transduction process, TIR1/AFB auxin receptors (TRANSPORT INHIBITOR RESPONSE1/AUXIN SIGNALING F-BOX), Aux/IAA transcriptional repressors (AUXIN/ INDOLE-3-ACETIC ACID) and ARF transcription factors (AUXIN RESPONSE FACTOR) are key elements regulating auxin signal transduction [82]. *SAUR* (*SMALL AUXIN UP RNA*) gene family is the largest class of auxin early response genes and widely exists in many plants [83]. The vast majority of *SAURs* gene sequences do not contain introns, and there are often one or more auxin response elements AuxREs in the promoter region [84, 85]. *GH3* gene family has been fully identified in *Arabidopsis thaliana*, and it is also the main auxin response gene [86]. Our results showed that the expression levels of 3 Aux/IAA transcriptional repressors and 7 auxin-response gene (Including *GH3*, *SAUR* and *Aux/IAA* genes) were significantly different between MS line and MR line (Fig. 6A). Furthermore, we also found that there are four key DEGs in the auxin biosynthesis pathway. Meanwhile, the content of IAA in MS line was lower than that in MR line at both the early uninucleate and binucleate stages (Fig. 6B). Previous studies have shown that low IAA content makes pollen mother cells starve, and the reduction of IAA content may be a precursor to pollen abortion [87]. In our study, the differential expression of auxin signal pathway related genes and auxin content in MS line were significantly lower than those in MR line. It is not difficult to infer that there is an important relationship between pollen abortion of MS line and auxin signal pathway.

Abscisic acid plays an important role in plant growth and development and abiotic stress response [88, 89]. The importance of abscisic acid (ABA) in anther development has been demonstrated previously. Exogenous ABA suppresses anther development and causes pollen abortion in tomato [90]; in the *ABSCISIC ACID-INSENSITIVE8* mutant, ABA insensitivity causes male sterility in *Arabidopsis* plants [91]; in the cytoplasmic male sterile cabbage lines, the microspore abortion could relate to the excessive ABA content in the anthers and leaves. The biosynthetic pathway of ABA in higher plants is mainly de novo synthesis in the plastids and cytoplasm. *NCED* gene family is indispensable for ABA synthesis pathway, which is with either 9-cis violaxanthin or 9-cis-neoxanthin as a substrate, and its expression level can reflect the level of endogenous ABA in plants [92]. Therefore, *NCED* gene regulates plant growth and development mainly by regulating the level of endogenous ABA in plants. A previous study showed that *AtNCED2* and *AtNCED3* are involved in regulating the development of Arabidopsis anthers [93]. In this study, *TraesCS5B02G375900* was identified as *NCED* gene, and RNA-seq results showed that it was up-regulated in the binucleate stage, which was consistent with qRT-PCR results (Fig. 7A, C). In the ABA signal transduction pathway, PYR/PYL/RCAR recognizes and binds ABA, removes the inhibition of PP2c on SnRK2s, and activates the downstream gene of ABA signal. ABA is first recognized by the intracellular receptor protein PYR/PYL/RCAR in plants [94]; In *Arabidopsis thaliana*, PP2C is a negative regulator in ABA core signal pathway, which is particularly important for plant stress adaptation, growth and development under non stress or stress conditions [95, 96]. Previous studies have shown that *SnRK2s* play an important role in plant osmotic stress response and ABA signal response [97]. *SnRK2s* can phosphorylate bZIP family transcription factors ABF1, ABF2/AREB1 and ABI5, and promote the table of ABA downstream response genes [98]. In this study, multiple DEGs are enriched into ABA signal transduction pathway (Fig. 7A). Among them, four DEGs related to PP2c function were up-regulated in the early uninucleate stage of MR line, but their expression in the binucleate stage of MS line was generally higher than that in MR line. Because PP2c has an inhibitory effect on SnRK2s, DEGs related to SnRK2s function show an opposite expression pattern with those related to PP2c. In addition, the gene (*TraesCS1A02G306300*) related to the regulation of ABA response gene is up-regulated in the early uninucleate stage of MR line and the binucleate stage of MS line. At the same time, quantitative analysis of plant hormones showed that ABA content was significantly higher in the early uninucleate stage of MR line than in MS line, but it was significantly higher in the binucleate stage of MS line than in MR line (Fig. 3B). Previous studies have shown that ABA can promote the early development of anthers [99], but too much ABA in the late stage of anther development is not conducive to the development of anthers and microspores [100]. It can be speculated that the down-regulation of ABA signal pathway related genes at the early uninucleate stage and the up-regulation of ABA signal pathway related genes at the binucleate stage are important factors for pollen abortion of MS line.

In a word, considering the pollen abortion of K-type CMS wheat MS(KOTS)-90-110 from the plant hormone level is a feasible scheme for exploring the mechanism of K-type CMS wheat pollen development. The analysis of the expression pattern of DEGs related to IAA and ABA signal pathways and the quantitative analysis of hormone content in the two stages of anther development of MS line and MR line showed that the two hormones had positive effects on microspore development at the early stage of anther development. However, at the late stage of microspore development, IAA still plays an active role in regulating microspore development, while ABA plays the opposite role. This is consistent with the research results of Tang et al. (2008) [101]. However, due to the complexity of the genome of allohexaploid wheat, the regulation mechanism of plant hormones on wheat pollen development needs to be further studied.

### The role of MAPK signal transduction pathway in the anther development of K-type CMS wheat

MAPK cascade pathway is a crucial signal transduction pathway, which plays an important role in various plant life activities. MAPK signal transformation pathway is involved in regulating plant growth and development, such as cytokinesis, root growth, flower organ development, grain size and number, stomatal development, embryonic development, etc. In addition, MAPK signaling pathway also plays a key role in regulating plant responses to stresses such as high salt, drought, extreme temperature, insects and pathogens. It responds to abiotic and biological stresses by regulating the stability of related proteins, plant hormone biosynthesis and signal transduction pathways [102]. Previous research showed that plant-pathogen interaction pathway may act as positive regulators in pollen development [103]. *AtMPK3*, *AtMPK4* and *AtMPK6* regulate plant-pathogen interaction by regulating the biosynthesis of plant antitoxins and plant hormones (such as ethylene, abscisic acid, etc.) [104]. The biosynthesis, transport and signal transduction of plant hormones are intricately related to MAPK signal, and some MAPKs control the biosynthesis or transport of hormones as upstream regulators. Other MAPKs are downstream and regulate hormone signal transduction. Taking *Arabidopsis* as an example, MAPKs are involved in the synthesis of jasmonic acid, auxin, ethylene and salicylic acid, as well as the signal transduction of auxin, ethylene, brassinolide, cytokinin, salicylic acid, abscisic acid and jasmonic acid [105]. In this study, many DEGs were enriched in MAPK signaling pathway (Fig. 5B). As shown by KEGG enrichment analysis, MAPK signaling pathway related DEGs are mainly up-regulated in the binucleate stage, but down-regulated in the early uninucleate stage (Fig. 8B). These DEGs are mainly involved in plant-pathogen interaction, ethylene signal pathway, ABA signal pathway and ROS metabolism pathway. ROS plays a significantly important role in plant growth and development, stress and response processes, seed germination, PCD and other physiological processes [106]. A large number of studies have shown that ROS provides energy for microspore development by regulating the PCD process of tapetum during anther tissue development. On the other hand, excessive accumulation of ROS will also lead to pollen abortion [107]. In the male sterile line IAMSL of wheat, because the levels of ascorbic acid and glutathione were higher than those of the maintainer line, the activity of the antioxidant system was reduced, resulting in excessive ROS, which ultimately led to microspore abortion [108]. The up-regulated expression of plant-pathogen interaction related DEGs in the binucleate stage of MS line may not be the reason for the abortion of MS line pollen, but the abnormal development of MS line anthers makes its pollen more susceptible to infection by pathogenic bacteria. In addition, DEGs in MAPK cascade pathway participate in ABA signal transduction pathway, which further indicated that abscisic acid plays an important role in K-type CMS wheat anther development.

The above results indicated that MAPK signal pathway in K-type CMS wheat anther regulates microspore development by participating in a variety of biochemical pathways, and the functions of related DEGs on microspore development and the regulatory network between them need to be further studied and verified.

### Alternative splicing may play an important role in the development of K-type CMS wheat anthers

 Accurate splicing of pre-mRNA is essential for plants to perform normal cytological functions and respond to genetic and environmental signals. Pre-mRNA splicing is a complex process catalyzed by spliceosomes based on the recognition of splicing sites. This process is accompanied by the binding and dissociation of different small nuclear ribonucleoproteins (snRNPs). Splicerosome is a macromolecular complex composed of 5 snRNAs (U1, U2, U4, U5 and U6) and more than 150 protein ribonucleoproteins (RNPs) [109]. Previous studies have shown that alternative splicing regulates the development of pollen during anther development [110]. For example, a histone H3K36 di- and tri-methylase (BrSDG8) associated with regulation of alternative splicing [111], is predominantly expressed as a non-functional isoform encoding a precision stop code on the tetrad stage of population development. In *Brassica rapa*, a dynamic intron retention program regulates the expression of several hundred genes during pollen meiosis [28]. In addition, many genes will ensure the normal development of pollen through alternative splicing when plants are under stress. Our results showed that 23 DEGs participate in the splicing body structure, and they are mainly up-regulated at the early uninucleate stage of MS line. It can be inferred that the gene that causes K-type CMS wheat pollen abortion may regulate the pollen development process by alternative splicing. In the future, further research on the mechanism of alternative splicing of wheat pollen development regulation genes will be a very feasible strategy to analyze the mechanism of pollen abortion.

### Possible transcriptome-mediated male sterility network in K-type CMS wheat

**Fig. 11:**
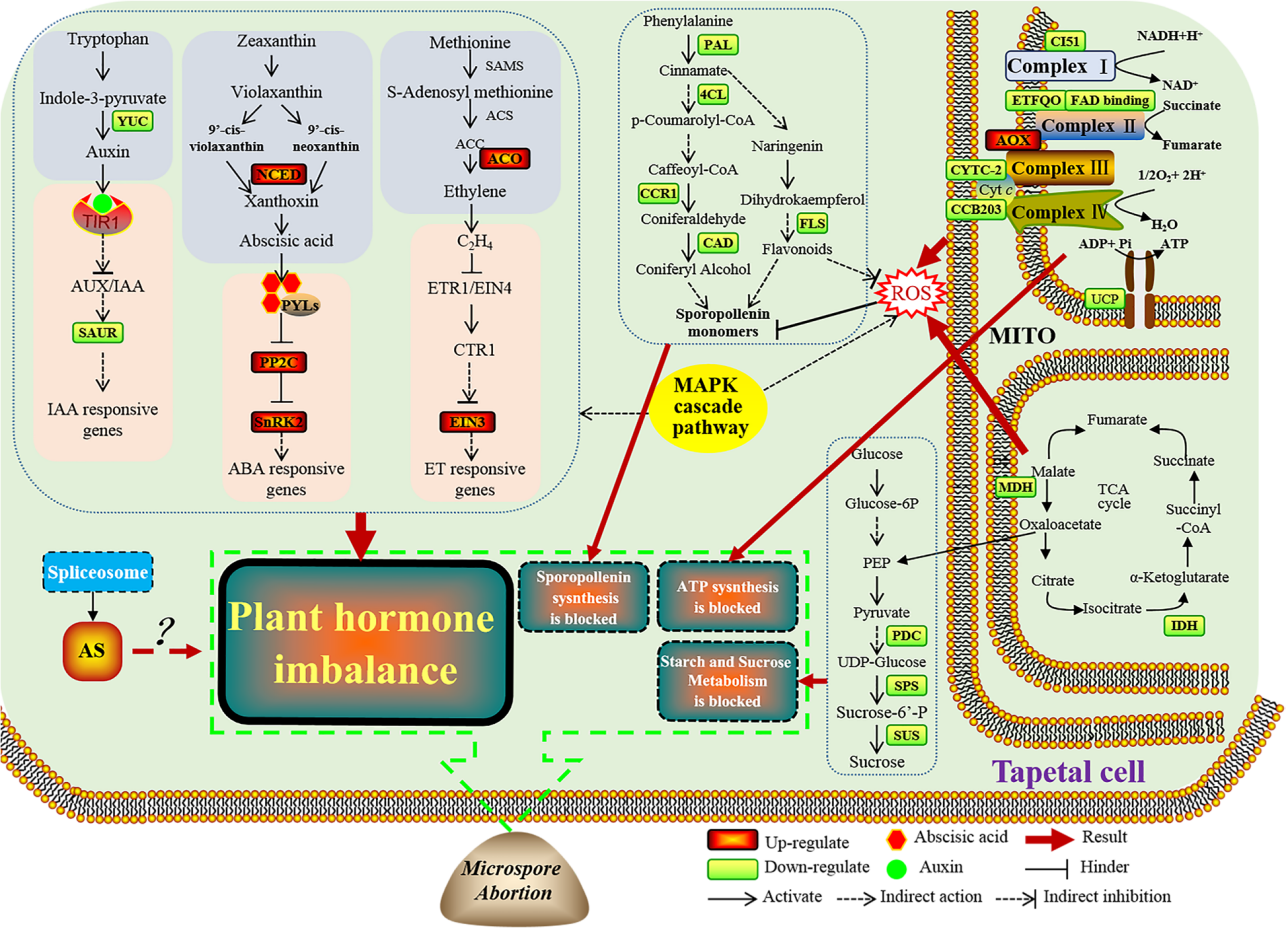
Possible transcriptome-mediated male sterility network in K-type CMS wheat. MITO, mitochondria; ROS, reactive oxygen species; CI51, NADH dehydrogenase; ETFQO, electron transfer flavoprotein alpha; FAD binding, FAD binding domain; CYTC-2, cytochrome c-2; CCB203, cytochrome c biogenesis orf203; AOX, alternative oxidase; UCP, uncoupling protein; IDH, isocitrate dehydrogenase; MDH, malate dehydrogenase; PDC, pyruvate decarboxylase; SPS, sucrose-phosphate synthase; SUS, sucrose synthase; FLS, flavonol synthases; PAL, phenylalanine ammonia-lyase; 4CL, 4-coumarate: CoA ligase; CCR1, cinnamoyl-CoA reductase 1; CAD, cinnamyl alcohol dehydrogenase; ACO, ACC oxidase; EIN3, ethylene insensitive 3; NCED, 9-cis epoxycarotenoid dioxygenases; PP2C, the type 2C protein phosphatases; SnRK2, sucrose non-fermenting-1-related protein kinase 2; YUC, key enzyme for auxin synthesis; SAUR, small auxin-up RNA. We used the software of Adobe Illustrator CS5 (Adobe, America) to draw this figure by ourselves

According to the KEGG cluster and MapMan analysis of DEGs, we analyzed the DEGs in the several important metabolic pathways (plant hormone signal transduction, MAPK signal transduction, the structure of spliceosome, starch and sucrose metabolism, phenylpropanoid and flavonoid biosynthesis), as well as considering determination of endogenous IAA and ABA content, cytological observation and above results, we propose a possible transcriptome-mediated male sterility network in K-type CMS wheat (Fig. 11). The tapetum cell provides sucrose, proteins, lipids, and sporopollenin to support the growth and development of the microspore via its degradation and secretion, it’s abnormal development may lead to microspore abortion. More and more studies have shown that plant male sterility is associated with mitochondrial genomes [112], and many CMS genes have been identified as mitochondrial electron transfer chain (mtETC) pathways [10]. In this study, a large number of DEGs related to the mtETC structure (CI51, ETFQO, FAD binding, CYTC-2, CCB203 and UCP) were downregulated in MS line. When the electron transport chain is suppressed, electrons will interact directly with oxygen molecules to produce ROS, and excess ROS will lead to abnormal PCD in the tapetum and male sterility. Mitochondrial uncoupling proteins (UCPs) are crucial for ATP biosynthesis and ROS balance. Knockdown of mitochondrial uncoupling proteins1 and 2 (AtUCP1 and 2) in *Arabidopsis thaliana* impacts vegetative development and fertility [113].

In addition, the upregulation of alternative oxidase (AOX) expression may be caused by the inhibition of cytochrome pathway, this is consistent with Karen’s research [114]. Previous studies have shown that defects in the TCA cycle may also lead to producing excessive amounts of ROS. We found that the expression levels of MDH and IDH in MS line were lower than those in MR line. We speculate that the above DEGs in MS line is likely to hinder ATP biosynthesis and lead to excessive accumulation of ROS, ultimately becoming one of the key factors for pollen abortion. Phenylalanine ammonia-lyase (PAL), 4-coumarate: CoA ligase (4CL), cinnamoyl CoA reductase (CCR) and cinnamyl alcohol dehydrogenase (CAD) are the key enzymes in the phenylpropanoid metabolic pathway. Additionally, flavonol synthase (FLS) is a key enzyme in flavonoid synthesis pathway. Downregulation of the expression of related genes encoding the above key enzymes and the excessive accumulation of ROS may lead to the blockage of sporopollenin synthesis. During anther development, flaws in sugar metabolism could make for male sterility [115, 116].PDC, SPS, and SUS are key components of the starch and sucrose metabolism pathway. Their downregulation leads to abnormalities in this pathway, which may ultimately lead to abnormal microspore development. More importantly, the key enzyme for auxin synthesis, YUCCA (YUC), is a key rate-limiting enzyme in the auxin synthesis pathway [117]. The downregulation of YUC expression may lead to a lower IAA content in MS line anthers than in MR line anthers, and the downregulation of SAUR expression may also block the IAA signal transduction pathway. Meanwhile, upregulated expression of NCED in ABA biosynthesis pathway and PP2C and SnRK2 in ABA signal transduction pathway. ACC Oxidase (ACO) is a key enzyme in the ethylene biosynthesis pathway, and its upregulation may increase the ethylene content in MS line anthers. Ethylene sensitive 3 (EIN3) can activate downstream genes involved in ethylene signal transduction [118], and it has transcriptional activation effects on *ACO* genes [119], thereby promoting ethylene synthesis. From this, it can be inferred that the upregulation of ethylene insensitive 3 (EIN3) expression further promotes the ethylene signal transduction pathway. Therefore, abnormalities in plant hormone biosynthesis and signal transduction pathways may lead to imbalances in hormone levels in MS line anthers, which is another important reason for K-type CMS wheat pollen abortion. In addition, the MAPK cascade pathway and the AS process also play an important role in this network. The MAPK cascade pathway regulates the balance of endogenous hormones in plants and actively acts on the ROS metabolic pathway. More importantly, the expression level of AS-related DEGs in MS line anthers was significantly higher than that in other samples at the early uninucleate stage. We speculate that alternative splicing events are another key factor leading to pollen abortion in MS line, but its mechanism of action remains to be further studied.

## Conclusions

By comparing the transcriptome data and the determination of plant hormone content of MS line and MR line anthers, we found that differences in gene expression of the plant hormone signal transduction and hormone imbalance were the main cause of the microspore abortion. Meanwhile, MAPK cascade pathway and alternative splicing may also play important regulatory roles in this process. In addition, we also identified differentially expressed genes related to energy metabolism, glucose metabolism, and sporopollenin synthesis pathways, which together constitute the regulatory network for K-type CMS microspore abortion. These findings provided intriguing insights for the molecular mechanism of microspore abortion in K-type CMS, and also give useful clues to identify the crucial genes of CMS in wheat.

### Supplementary Information

**Additional file 1: Figure S1. **FPKM violin distribution analysis. The horizontal axis indicates different samples, and the vertical axis indicates corresponding sample FPKM. **Figure S2. **Correlation analysis between biological replicates. The horizontal axis and vertical axis represent each sample. The color represents the correlation coefficient, the bluer the color, the higher the correlation, and the whiter the color, the lower the correlation. **Figure S3. **Volcano plots showing the expression levels of every gene in the core set of shared DEGs.Note that the red dots represent up-regulation and the green dots represent down-regulation.

**Additional file 2: Supplementary Table S1.** Differentially expressed genes in MS line VS MR line(MSu vs MRu, MSb vs MRb, MSu vs MSb, MRu vs MRb). **Supplementary Table S2.** GO terms enriched for "MS line-VS-MR line" DEGs. **Supplementary Table S3.** KEGG pathways enriched for "MS line-VS-MR line" DEGs. **Supplementary Table S4.** ManMap pathways enriched for "MS line-VS-MR line" DEGs. **Supplementary Table S5.** qRT-PCR primer sequences for qPCR validation of plant hormone related DEGs in RNA sequencing data. **Supplementary Table S6.** The wheat-specific Taes_AFFY_0709 mapping file" Taes_AFFY_0709".

## Data Availability

All data generated in this study are available within the paper and its additional files. The raw data in this study have been deposited in the China National Center for Bioinformation, under accession numbers CRA011155, that are accessible at https://ngdc.cncb.ac.cn/gsa/browse/CRA011155.

## Abbreviations

CMS: Cytoplasmic male sterility
DEGs: Differentially expressed genes
GO: Gene Ontology
KEGG: Kyoto Encyclopedia of Genes and Genomes
ORF: Open reading frame
ATP: Adenosine-Triphosphate
PCD: Programmed cell death
ROS: Reactive oxygen species
MAPK: Mitogen-activated protein kinase
AS: Alternative splicing
ES: Exon skipping
IR: Intron retention
A5SS: Alternative 5’ splice sites
A3SS: Alternative 3’ splice sites
MEs: Mutually exclusive exons
SEM: Scanning electron microscopy
