# Supplementary material for: Transcriptomics reveals a core transcriptional network of K-type cytoplasmic male sterility microspore abortion in wheat (Triticum aestivum L.)
Source: BMC Plant Biol. 2023 Dec 6;23:618. doi: 10.1186/s12870-023-04611-2 (PMC10698923; doi:10.1186/s12870-023-04611-2)
Supplement: Supplementary file 1 — Additional file 1: Figure S1. FPKM violin distribution analysis. The horizontal axis indicates different samples, and the vertical axis indicates corresponding sample FPKM. Figure S2. Correlation analysis between biological replicates. The horizontal axis and vertical axis represent each sample. The color represents the correlation coefficient, the bluer the color, the higher the correlation, and the whiter the color, the lower the correlation. Figure S3. Volcano plots showing the expression levels of every gene in the core set of shared DEGs.Note that the red dots represent up-regulation and the green dots represent down-regulation. [file 12870_2023_4611_MOESM1_ESM.pdf]

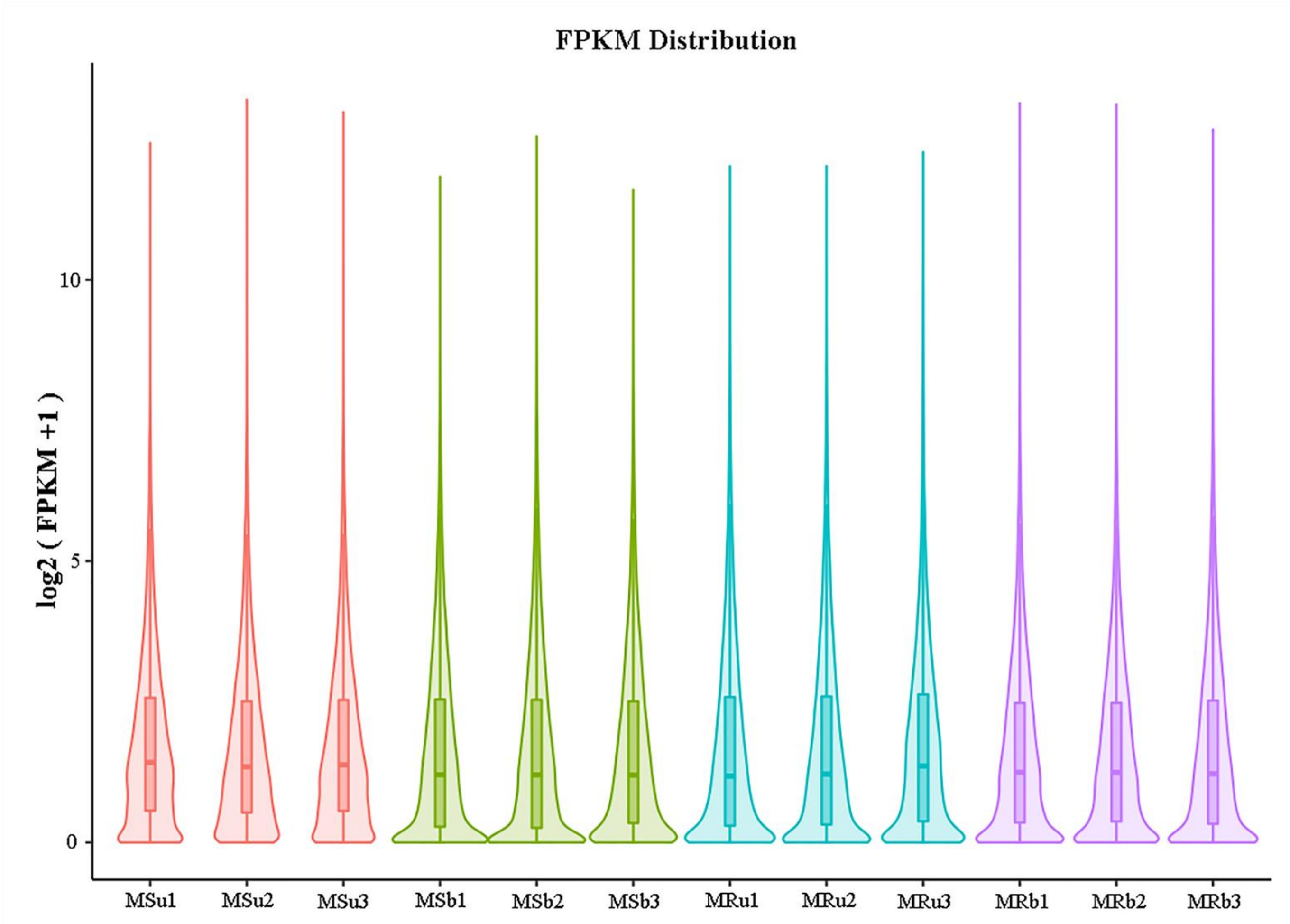

Figure S1. FPKM violin distribution analysis. The horizontal axis indicates different samples, and the vertical axis indicates corresponding sample FPKM.

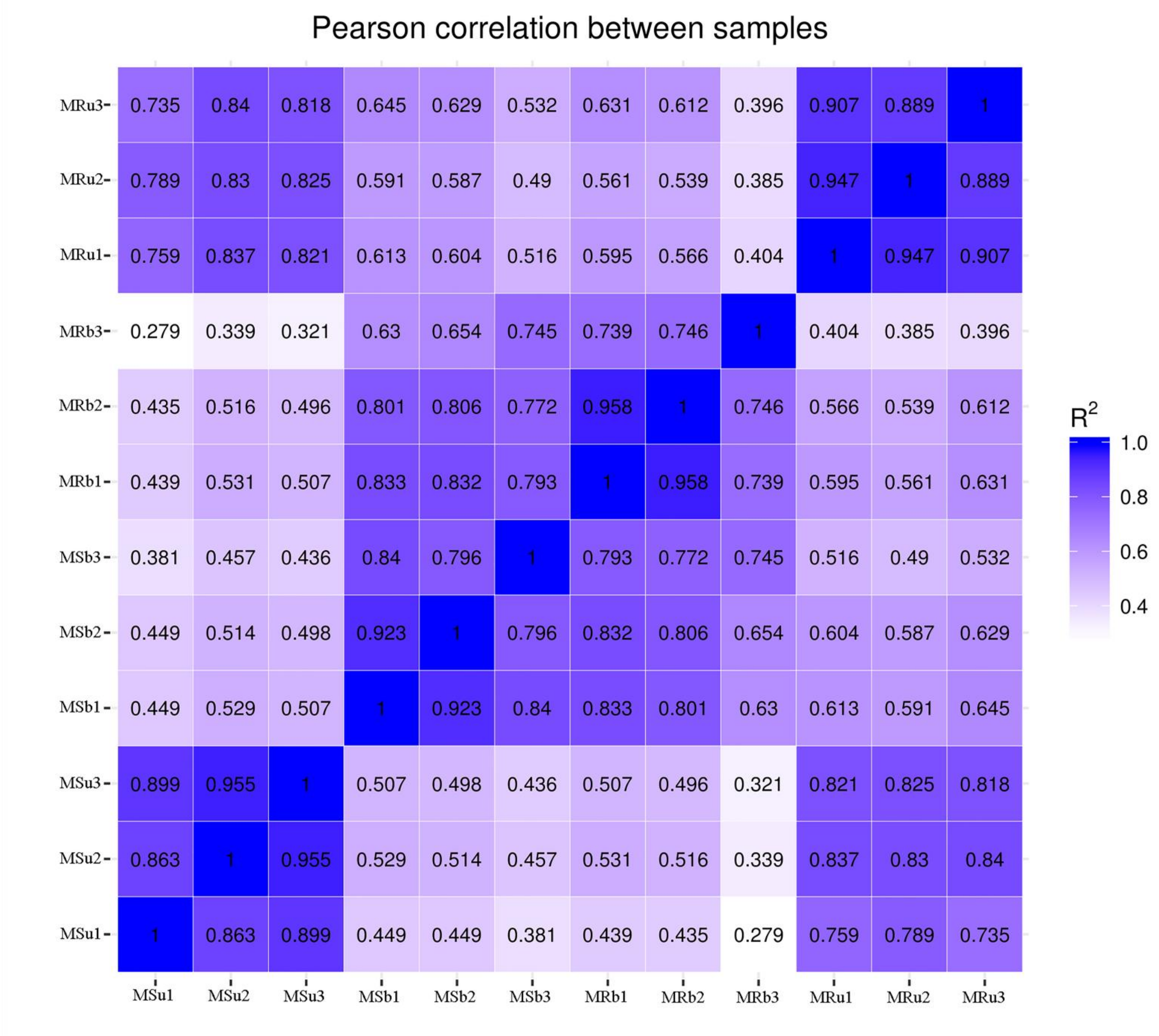

Figure S2. Correlation analysis between biological replicates. The horizontal axis and vertical axis represent each sample. The color represents the correlation coefficient, the bluer the color, the higher the correlation, and the whiter the color, the lower the correlation.

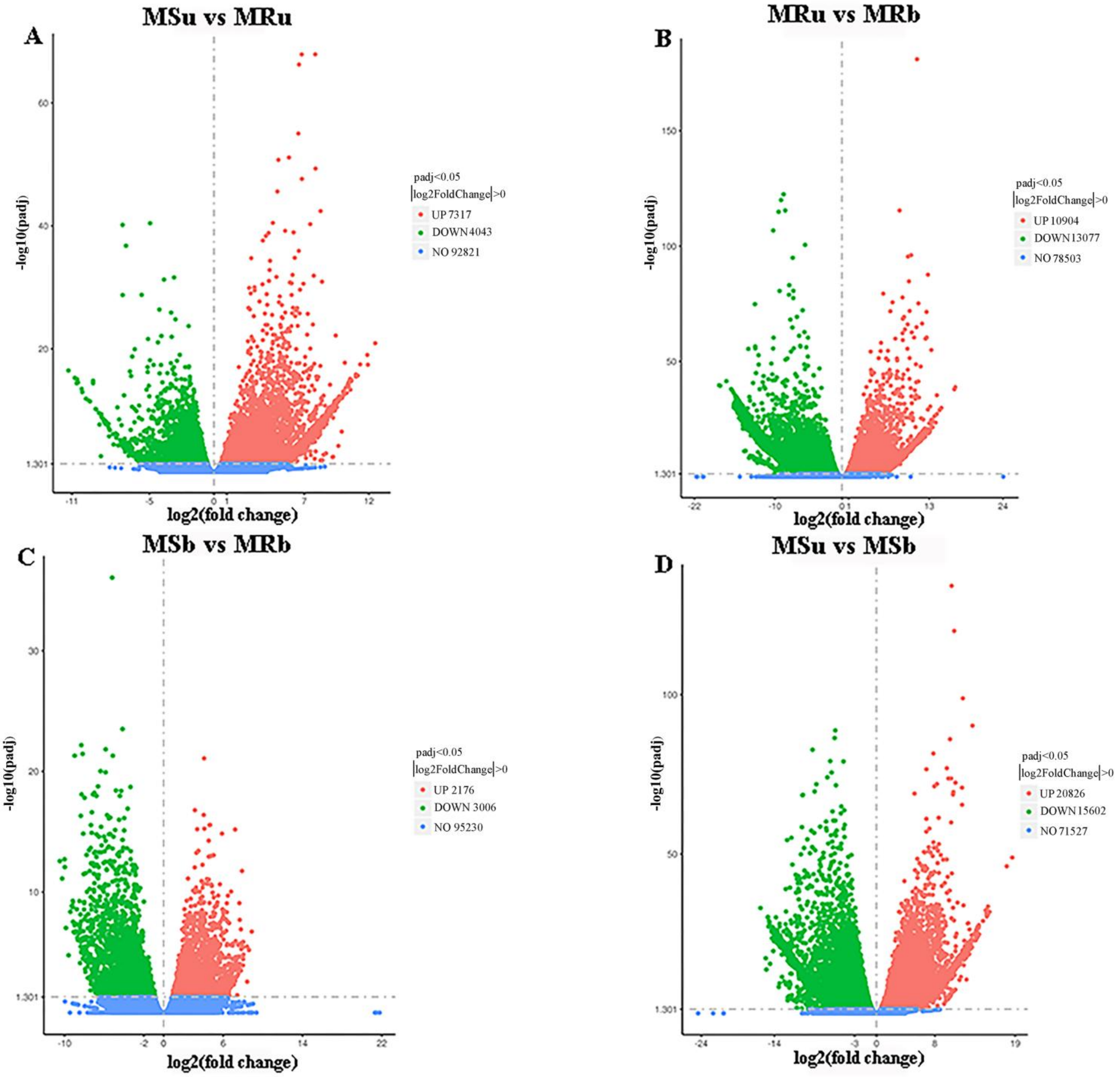

Figure S3 Volcano plots showing the expression levels of every gene in the core set of shared DEGs.Note that the red dots represent up-regulation and the green dots represent down-regulation.
